# Metal–organic framework-based wearable electrochemical glucose sensors for sweat analysis: materials design, sensing mechanisms, and future perspectives

**DOI:** 10.1039/d6ra06028e

**Published:** 2026-09-25

**Authors:** Parkkavi Krishnamoorthy, Ajay Rakkesh Rajendran

**Affiliations:** a Functional Nano-Materials (FuN) Laboratory, Department of Physics and Nanotechnology, Faculty of Engineering and Technology, SRM Institute of Science and Technology Kattankulathur – 603203 TN India ajayr1@srmist.edu.in ajayrakkesh@gmail.com; b Department of Chemistry, SRM Institute of Science and Technology Kattankulathur – 603203 TN India

## Abstract

The global prevalence of diabetes mellitus has intensified the demand for continuous, reliable, and patient-friendly glucose monitoring technologies. Conventional blood-based glucose testing remains invasive and unsuitable for continuous monitoring, motivating extensive research into non-invasive wearable sensing platforms. Sweat has emerged as an attractive biofluid for glucose monitoring due to its accessibility and compatibility with skin-interfaced devices; however, the low glucose concentration, complex matrix, and dynamic secretion rates pose significant analytical challenges. Metal–organic frameworks (MOFs), a class of crystalline porous materials constructed from metal nodes and organic linkers, offer unique opportunities to address these challenges owing to their exceptionally high surface areas, tunable pore architectures, and versatile chemical functionalities. Recently, MOF-based electrochemical glucose sensors have shown considerable promise, particularly when integrated into flexible and wearable systems for sweat analysis. This review critically examines recent advances in MOF-enabled wearable electrochemical glucose sensors, with emphasis on material design principles, enzymatic and non-enzymatic sensing mechanisms, device integration strategies, and key performance metrics. Current limitations related to conductivity, stability, biofouling, and physiological variability are discussed, along with emerging strategies to overcome these barriers. Finally, this review outlines future directions toward intelligent, clinically relevant MOF-based wearable glucose monitoring systems.

## Abstract 2

From molecular-scale pore engineering to flexible, skin-integrated biosensing-unlocking sweat as a non-invasive window into metabolic health.

## Graphical abstract

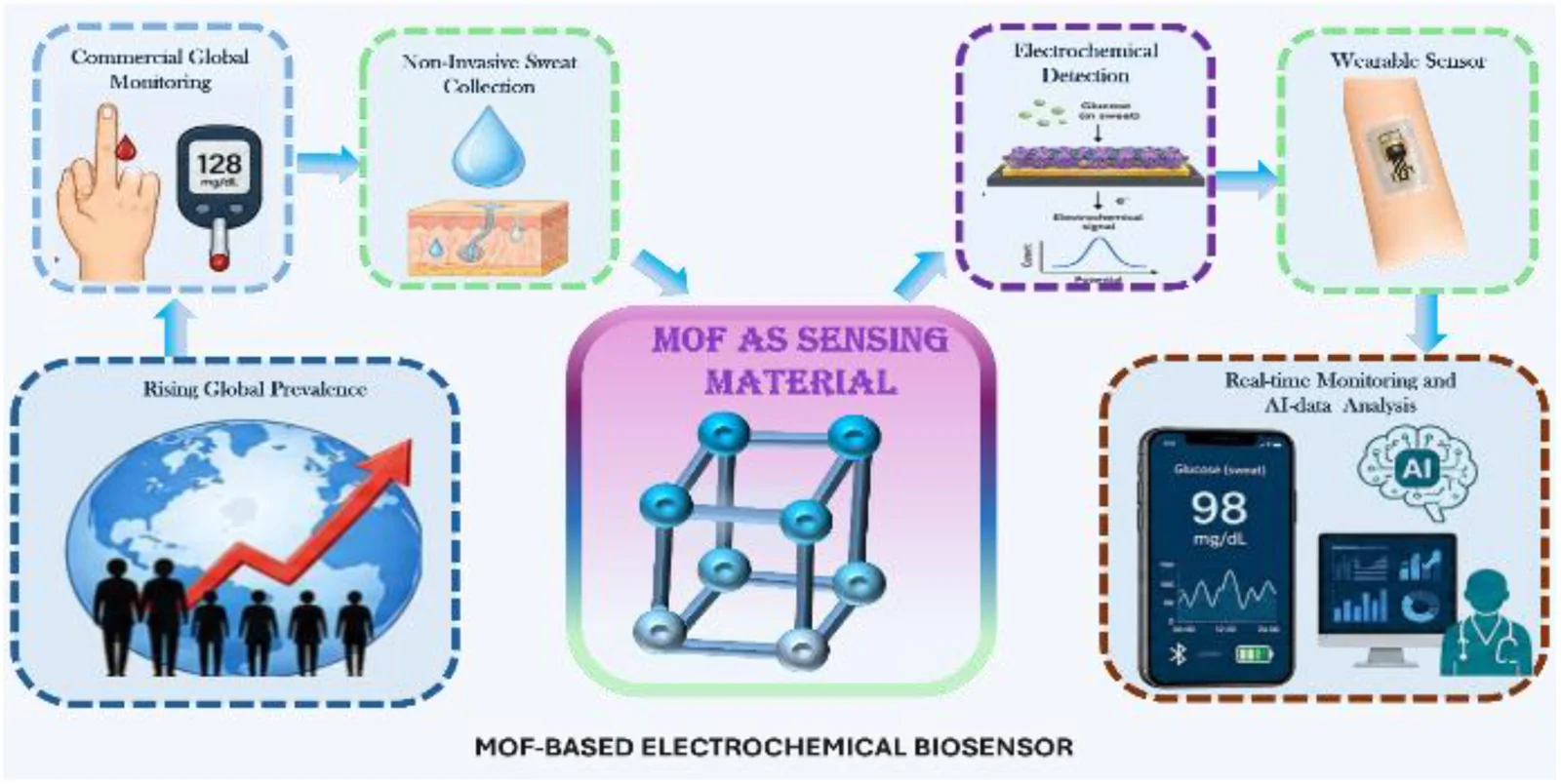

## 1. Introduction

Diabetes mellitus is one of the most prevalent chronic diseases worldwide, affecting hundreds of millions of individuals and imposing a substantial burden on healthcare systems. Effective glycemic control is essential to prevent both acute complications and long-term sequelae such as cardiovascular disease, neuropathy, nephropathy, and retinopathy. Central to diabetes management is frequent glucose monitoring, which traditionally relies on finger-prick blood sampling. Although accurate, this approach is invasive, uncomfortable, and poorly suited for continuous monitoring, often leading to reduced patient compliance.^1–5^

The limitations of conventional blood glucose monitoring have driven significant interest in alternative sensing strategies that are minimally invasive or entirely non-invasive. Wearable biosensors capable of continuously tracking physiological biomarkers have emerged as a promising solution, enabled by advances in flexible electronics, materials science, and data analytics. Among the various biofluids explored for non-invasive glucose sensing, sweat has attracted particular attention, alongside interstitial fluid, saliva, and tears.^6–10^ Sweat can be collected non-invasively, continuously, and without the need for microneedles or implantable devices, making it especially attractive for long-term wearable applications.^6,7,11^

Despite these advantages, sweat-based glucose sensing presents formidable challenges. Glucose concentrations in sweat are typically two to three orders of magnitude lower than in blood, often in the micromolar range. Additionally, sweat composition varies with physiological state, sweat rate, environmental conditions, and individual differences, complicating quantitative analysis. These challenges necessitate sensing materials and device architectures that combine ultrahigh sensitivity, excellent selectivity, mechanical robustness, and long-term stability.^6,11,12^

Numerous glucose-sensing technologies have been explored over recent decades, including acoustic, magnetic, thermal, optical, and electrochemical transducers. Compared to other analytical techniques, electrochemical biosensors (ECBs) are among the most promising analytical tools due to their cost-effectiveness, rapid response times, and high sensitivity.^1,13–18^ Their miniaturised design facilitates on-site and *in situ* measurements, making them ideal for various applications, from monitoring bioanalytes to detecting environmental pollutants. Recent advancements in wearable and point-of-care sensors highlight their utility for remote, continuous monitoring. A critical factor in ECBS performance is electrode material selection. Consequently, researchers have explored various nanomaterials, polymers, and hybrid structures. However, challenges remain in long-term stability in real-world environments and in balancing high performance with cost-effective bulk production.^12,19–21^

Metal–Organic Frameworks (MOFs) have emerged as a versatile class of porous crystalline materials for the development of advanced chemical and biological sensing platforms. Structurally, MOFs are constructed by coordinating metal ions or metal-containing clusters with organic ligands, producing ordered one-, two-, or three-dimensional frameworks. Unlike conventional sensing materials, including metal oxides, noble metals, carbon-based materials, conducting polymers, and molecularly imprinted polymers, MOFs offer great control over their composition, pore environment, metal nodes, organic linkers, and functional groups.^19,22–26^ This molecular-level tunability enables the rational design of MOF-based sensing platforms with specialised structures and accessible active sites for specific interactions with target analytes. As a result, MOFs have attracted considerable attention for detecting ions, gases, small molecules, metabolites, proteins, nucleic acids, and other biologically relevant species.^27–30^

The fundamental sensing performance of MOFs stems from interactions between the target analyte and the functional framework. Depending on the sensing platform and signal transduction strategy, analyte recognition can induce changes in electrochemical, optical, electrical, or mass-sensitive signals. In electrochemical sensing, the analyte may interact directly with catalytically active metal centres, functional organic ligands, or immobilised recognition elements, resulting in changes in electron-transfer kinetics, faradaic current, charge transfer resistance, or electrode potential. For redox-active MOFs, the metal nodes themselves can participate in reversible redox reactions, thereby facilitating the electrocatalytic conversion of electroactive analytes. In addition, the porous architecture of MOFs promotes the diffusion and preconcentration of analyte molecules near electroactive sites, thereby increasing the probability of interfacial electron-transfer reactions. These mechanisms form the basis for amperometric, voltammetric, potentiometric, and impedimetric MOF-based sensing strategies.^31–33^ The sensing mechanism of MOFs can also involve selective host–guest interactions. The ordered pores and chemically tunable internal surfaces can preferentially adsorb molecules according to their molecular size, shape, polarity, charge, or coordination affinity. Functional groups present in the organic ligands or introduced through post-synthetic modification can further provide specific interactions, including hydrogen bonding, electrostatic attraction, π–π interactions, and coordination bonding. Such interactions can improve analyte recognition and discrimination in complex chemical and biological environments. In biosensing applications, the large surface area and porous structure of MOFs additionally provide an effective matrix for the immobilisation of biomolecules, including enzymes, antibodies, aptamers and nucleic acids, while maintaining close contact between the biorecognition element and the signal-transduction interface.^25,34–37^ Several intrinsic characteristics make MOFs particularly attractive for sensing applications. (i) Their exceptionally high surface area and hierarchical porosity provide abundant accessible sites for analyte adsorption and molecular recognition. (ii) Their pore size and chemical functionality can be adjusted through the selection of different metal centres and organic linkers, allowing the sensing interface to be tailored for specific target molecules. (iii) Many MOFs contain coordinatively unsaturated metal sites or redox-active metal centres that can participate directly in catalytic or electrochemical reactions. (iv) MOFs can be readily integrated with conductive materials, including carbon nanomaterials, conducting polymers, metallic nanoparticles, and other two-dimensional materials, to generate synergistic sensing platforms with improved charge transport and catalytic activity. These structural and functional advantages distinguish MOFs from many conventional porous materials and make them highly promising for the construction of sensitive and selective chemical and biological sensors.^38–40^ In chemical sensing, MOF-based materials have been widely investigated for the detection of metal ions, toxic pollutants, gases, pesticides, antibiotics, and other environmentally relevant analytes. Their importance arises not only from their high adsorption capacity but also from their ability to convert molecular recognition into measurable signals.^41–43^ Similarly, in biological sensing, MOFs have shown significant potential for the detection of physiologically relevant biomolecules and biomarkers. Their porous frameworks can facilitate analyte enrichment, their active metal centres can promote electrocatalytic reactions, and their large surface area can support a high loading of biological recognition elements. These characteristics are particularly beneficial for detecting low-abundance analytes, where efficient mass transport, signal amplification, and selective molecular recognition are required.^20,44–46^ Despite these advantages, pristine MOFs also exhibit several limitations that should be considered in sensor design. Many conventional MOFs possess relatively low intrinsic electrical conductivity, which can limit electron transport during electrochemical measurements. Furthermore, structural degradation or instability may occur under highly acidic, alkaline, or aqueous conditions, depending on the strength of metal–ligand coordination.^35,47,48^ To overcome these challenges, considerable research has focused on conductive MOFs, bimetallic MOFs, MOF composites, and MOF-derived materials. The incorporation of conductive carbon materials, metallic nanoparticles, conductive polymers, or secondary metal species can improve electrical conductivity, catalytic activity, structural stability, and the number of accessible active sites.^49–55^ Therefore, the transition from pristine MOFs to rationally engineered MOF-based composites represents an important strategy for achieving high-performance chemical and biological sensing platforms. Overall, the combination of structural tunability, high porosity, abundant active sites, molecular recognition capability, and ease of functionalization has established MOFs as an important materials platform for next-generation sensing technologies. These features are particularly relevant to electrochemical biosensing, where the sensing materials must simultaneously facilitate analyte transport, molecular recognition, catalytic conversion, and efficient electron transfer. However, the translation of these advantageous properties into practical sensing devices requires careful control of framework composition, conductivity, stability, electrode integration, and interfacial architectures. This is especially important for emerging wearable biosensors, where the sensing material must maintain analytical performance under mechanical deformation and operate reliably in complex biofluids. Moreover, the rational integration of MOF-based materials into wearable electrochemical platforms offers an important pathway toward non-invasive, continuous monitoring of biologically relevant analytes such as glucose.^21,46,49,56^

This review provides a comprehensive and critical overview of wearable electrochemical glucose sensors based on MOFs for sweat analysis. We discuss the fundamental aspects of sweat glucose sensing, the unique advantages of MOFs as functional sensing materials, recent progress in enzymatic and non-enzymatic MOF-based sensors, strategies for wearable integration, and key challenges that must be overcome for clinical translation.

## 2. Sweat as a biofluid for glucose monitoring

### 2.1 Composition and physiological relevance of sweat

Human sweat is an aqueous biofluid secreted primarily by eccrine sweat glands. It contains a wide range of analytes, including electrolytes (Na^+^, K^+^, Cl^−^), metabolites (glucose, lactate, urea), amino acids, and trace biomolecules. The concentration of glucose in sweat typically ranges from 1 to 100 µM, depending on individual physiology, sweat rate, and metabolic state. While sweat glucose levels do not directly mirror blood glucose concentrations, several studies have demonstrated a correlation between sweat glucose trends and blood glucose fluctuations under controlled conditions.^11,57,58^

### 2.2 Advantages and challenges of sweat-based sensing

Sweat-based glucose sensors have gained significant attention due to their non-invasive nature, painless operation and compatibility with wearable devices for continuous glucose monitoring. Unlike traditional blood-based methods, these sensors enable real-time health monitoring through skin-mounted platforms, improving patient comfort and compliance. However, several challenges limit their practical application.^59–61^ Sweat glucose occurs at considerably lower concentrations than blood glucose and is influenced by the dynamic composition of the sweat matrix. Furthermore, electroactive and biologically relevant species, including uric acid, ascorbic acid, and lactate, can interfere with glucose detection and reduce analytical selectivity.^62^ Variation in sweat secretion rate can additionally influence analyte dilution, transport, evaporation, and residence time at the sensing interface, leading to variability in the measured glucose concentration.^63–65^ A major physiological challenge is the relationship between blood glucose (BG) and sweat glucose (SG). Although temporal variations in SG may broadly follow changes in BG, the two signals are not necessarily quantitatively equivalent or synchronised. Glucose must be transported from the vascular compartment through the interstitial environment and eccrine sweat glands before reaching the skin surface. Consequently, changes in BG may appear in sweat after a physiological delay. Blood-to-sweat glucose delays of approximately 5–30 minutes have frequently been reported; however, the magnitude of this delay is not constant and may vary with sweat rate, exercise, hydration status, anatomical location, and individual physiology. Studies have also shown that glucose may take approximately 10 min to move from the blood to sweat. In addition, the ratio of sweat glucose to blood glucose can vary considerably with sweat rate, indicating that sweat secretion conditions strongly affect the interpretation of sweat glucose measurements. The variability of the BG–SG relationship further indicates that sweat glucose should not be considered a direct quantitative substitute for blood glucose. Physiological variables such as physical activity, temperature, hydration, food intake, metabolic state, and sampling location may introduce both intra- and inter-individual differences in sweat composition. Therefore, calibration approaches based on a fixed blood-to-sweat conversion factor or a constant physiological delay are unlikely to be universally applicable.^66–68^ Multiparameter wearable systems that simultaneously monitor glucose and physiological variables such as sweat rate, pH, temperature, and physical activity may improve the interpretation of SG signals. In addition, Machine-learning (ML) approaches can integrate these parameters to establish individualised calibration models and improve glucose trend prediction. Dynamic time warping (DTW) may further address temporal mismatch by aligning BG and SG profiles with variable physiological delays. Although ML and DTW-based approaches require further validation using larger longitudinal human datasets, their integration offers a promising strategy for dynamic and personalised sweat-glucose calibration.^64,69–72^

With this framework, advanced sensing materials are required to provide sufficiently sensitive and selective primary glucose measurements. Metal–organic frameworks (MOFs) are promising candidates because their high surface area, tunable pore environments, and redox-active metal centres can provide abundant catalytic sites for glucose oxidation. Their pore structure can also facilitate selective analyte transport, while integration with conductive carbonaceous materials, polymers, or other nanostructures can improve charge transfer kinetics and overcome the relatively poor intrinsic conductivity of many pristine MOFs. Accordingly, MOF-based sensing interfaces can primarily address analytical limitations such as sensitivity, electrocatalytic activity, selectivity, and response rate, whereas multiparameter sensing and computational calibration can address physiological variability and the complex BG–SG relationship. The integration of rationally engineered MOFs with wearable platforms and data-driven calibration therefore represents a promising direction for next-generation sweat glucose monitoring.^1,73,74^

## 3. Metal–organic frameworks: properties relevant to electrochemical sensing

### 3.1 Structural and chemical tunability

MOFs are distinguished by their exceptionally high surface areas (often exceeding 1000 m^2^ g^−1^), along with well-defined pore structures and modular chemistry. Selecting appropriate metal nodes and organic linkers enables precise control over MOFs, which can be engineered with specific pore sizes, surface functionalities, and chemical affinities.^75,76^ Therefore, the pore environment can be engineered to match the molecular dimensions of glucose, thereby promoting selective adsorption through size-selective confinement and host–guest interactions. In addition, incorporation of functional groups such as –NH_2_, –OH, –COOH, or sulfonic groups within the organic linkers can enhance hydrogen bonding and electrostatic interactions with glucose molecules. This tunability enables the selective adsorption of glucose molecules and facilitates interaction with catalytic sites.^37,77–80^ Overall, the structural and chemical tunability of MOFs offers a versatile platform for designing advanced sensing materials, enabling efficient molecular recognition and catalytic interaction with target molecules. This adaptability makes MOFs highly promising candidates for next-generation biosensing and diagnostic applications.

### 3.2 MOFs as functional platforms for glucose sensing

In electrochemical glucose sensors, MOFs can play multiple roles:

Metal–organic frameworks (MOFs) are a class of crystalline porous materials formed by the coordination of metal ions or metal clusters with organic ligands to create highly ordered three-dimensional structures. As illustrated in Fig. 1, a schematic representation of a metal–organic framework. Due to their exceptional characteristics, such as high surface area, tunable pore structures, and versatile chemical functionality, MOFs have attracted attention for various emerging platforms, including gas storage, catalysis, drug delivery, and electrochemical sensing. In recent years, MOF-based materials have attracted attention in the electrochemical sensing field because of their structural properties, which enable efficient interactions with analyte molecules and facilitate catalytic reactions at the electrode surface. The major advantage of MOFs is their large surface area and tunable porosity, which provide abundant active sites for analyte adsorption and enable electrochemical reactions. The pore size and internal surface chemistry of MOFs can be precisely controlled by careful selection of metal nodes and organic linkers. Hence, tunability enables MOFs to selectively interact with specific molecules and thereby improves the sensitivity and selectivity of the electrochemical sensing strategy. Furthermore, the interconnected pore networks within the MOFs facilitate the diffusion of analyte molecules from the bulk solution to the active sites, thereby promoting overall sensing performance.^77,79,81^ MOFs are widely used in enzyme immobilization matrices and in enzymatic glucose sensors. However, enzymes are sensitive to environmental conditions and may lose their activity when exposed to unfavourable conditions such as high temperature or extreme pH. Therefore, the porous framework provides a protective structural environment that can encapsulate or immobilise enzymes, thereby protecting biomolecules from denaturation.^82,83^ In addition to enzymatic sensing, MOFs can also function as electrocatalysts, particularly in non-enzymatic sensing, in which the non-enzymatic sensor eliminates the need for biological enzymes and instead relies on direct electrochemical oxidation of glucose at the electrode surface. MOFs consist of metal ions or clusters, which may be noble or non-noble transition metals, including (Ni, Co, Cu, Fe, Pt, Ag or Au) has catalytic activity that can promote glucose oxidation. These metal centres act as redox-active sites that can facilitate electron transfer during the electrochemical reaction.^84^ As a result, MOF-based electrocatalysts can significantly improve the sensitivity, stability and detection limits of a non-enzymatic sensor. Mass transport facilitates the movement of analyte molecules from the bulk solution to the electrode surface, where electrochemical reactions occur. The highly porous and interconnected channels within MOFs enable rapid diffusion of analyte molecules to the catalytic sites. This property improves the kinetics of electrochemical reactions and reduces the response time of sensors. Efficient mass transport is essential in biosensing, enabling efficient diffusion of analytes.^75,85^ MOFs can also serve as structural scaffolds for the formation of composite materials. Their well-defined porous architecture can be combined with various conductive materials such as graphene, carbon nanotubes, metal nanoparticles and conductive polymers. These composite systems integrate with MOFs and exhibit increased surface area, increased catalytic activity and also exhibit excellent electrical conductivity of MOFs. As a result, MOF-based supporting composite formation with conductive materials often enhances the significant improvement of electrochemical performance compared to pristine MOFs.^86,87^ Despite their numerous advantages, however, most pristine MOFs exhibit low intrinsic electrical conductivity. The poor conductivity arises from the limited overlap of electronic orbitals between metal nodes and organic ligands, resulting in insufficient electron transfer efficiency. In order to tackle these issues, various strategies have been proposed, such as MOF-based composites, doping with conductive nanoparticles, or necessitating careful material and device design to ensure efficient electron transport.

**Fig. 1:**
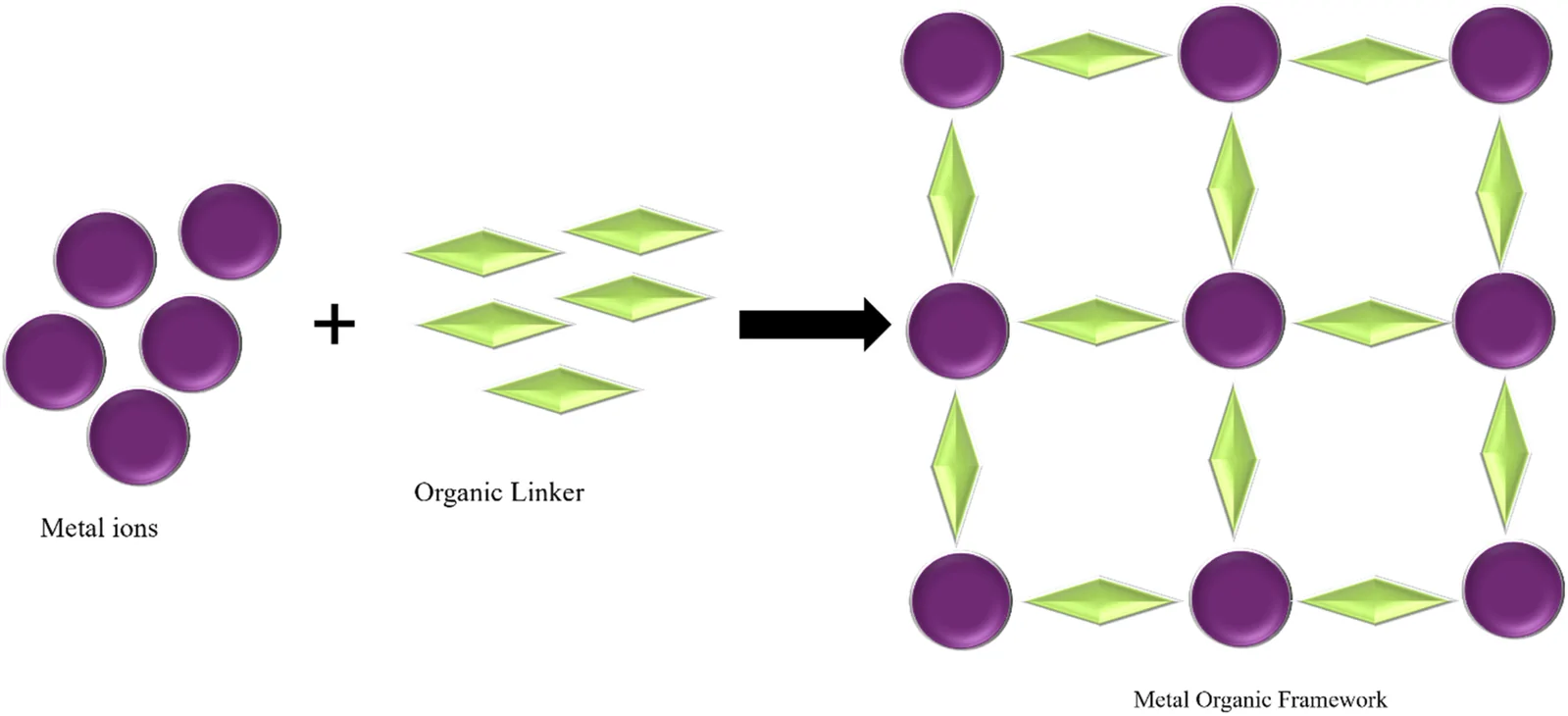
Schematic illustration of metal–organic framework: formation of MOFs through the coordination of metal ions and organic linkers. The self-assembly process generates a highly ordered porous network structure.

## 4. Evolution of MOF-based electrochemical glucose sensing mechanisms

Electrochemical transduction-based glucose sensors have achieved remarkable success because of their low fabrication cost, reliable analytical performance, and ease of operation, making them highly suitable for routine diabetes management. In these sensors, an electrical parameter such as current or voltage is applied as the input, while variations in current, voltage, or resistance are monitored to determine the glucose concentration^88^ as shown in Fig. 2, the first electrochemical glucose biosensor was introduced by Leland C. Clark in 1963 using a glucose oxidase (GOx)-modified platinum electrode, marking the arrival of the first generation of glucose sensors. The sensing mechanism depends on the enzymatic oxidation of glucose by GOx in the presence of oxygen, producing gluconic acid and hydrogen peroxide. The generated hydrogen peroxide is subsequently oxidised at the Pt electrode, producing an electrical signal proportional to the glucose concentration.^14,82,89,90^ Further advancements led to the replacement of GOx with glucose dehydrogenase (GDH), which employs cofactors such as pyrroloquinoline quinone (PQQ) or flavin adenine dinucleotide (FAD) instead of molecular oxygen as the electron acceptor. Consequently, GDH-based biosensors can operate at lower detection potentials and are less affected by fluctuations in dissolved oxygen levels. Despite these advantages, GDH-based systems face challenges related to cofactor stability and enzyme performance, limiting their widespread application compared with the GOx-based sensor. To overcome the limitations associated with oxygen-dependent electron transfer, second-generation glucose sensors were developed. These sensors employ artificial redox mediators, including methylene blue, indigo disulfonate, ferrocenemethanol, and benzyl viologen, to facilitate electron transfer from enzyme active sites to the electrode surface. The use of mediators enhances electron transfer efficiency and reduces dependence on oxygen; however, it introduces additional challenges, such as competition between oxygen and the mediator for electrons and the need for highly stable mediators capable of undergoing repeated redox cycling during continuous operation. The drawbacks associated with mediator-based systems subsequently motivate the development of third-generation glucose biosensors. These sensors are based on direct electron transfer (DET) between the enzyme and the electrode, which leads to the elimination of both oxygen and artificial mediators, whereas third-generation biosensors give improved selectivity and a simplified sensing mechanism; they still use biological enzymes, whose instability remains a major complication. Enzymes are susceptible to denaturation and chemical degradation during fabrication, storage, and operation, which can affect sensor accuracy, reproducibility and long-term performance. To resolve these problems, extensive efforts have been directed toward the development of non-enzymatic glucose biosensors, commonly referred to as fourth-generation glucose sensors.^91^ These systems utilise nanomaterials with enzyme-mimicking catalytic properties, known as nanozymes, to promote the direct electrooxidation of glucose without the use of biological enzymes. This approach enhances sensor stability, durability and operational reliability. Across all generations of glucose biosensors, advanced nanomaterials play major roles as electron-transfer channels, enzyme immobilisation matrices, and catalytic platforms. Among the various nanomaterials exhibited, Metal–Organic Frameworks (MOFs) have attracted significant attention because of their exceptionally high surface areas, tunable pore structures, versatile structural stability, and favourable electrochemical properties. These unique characteristics make MOFs a highly promising material for the design and development of next-generation glucose technologies.

**Fig. 2:**
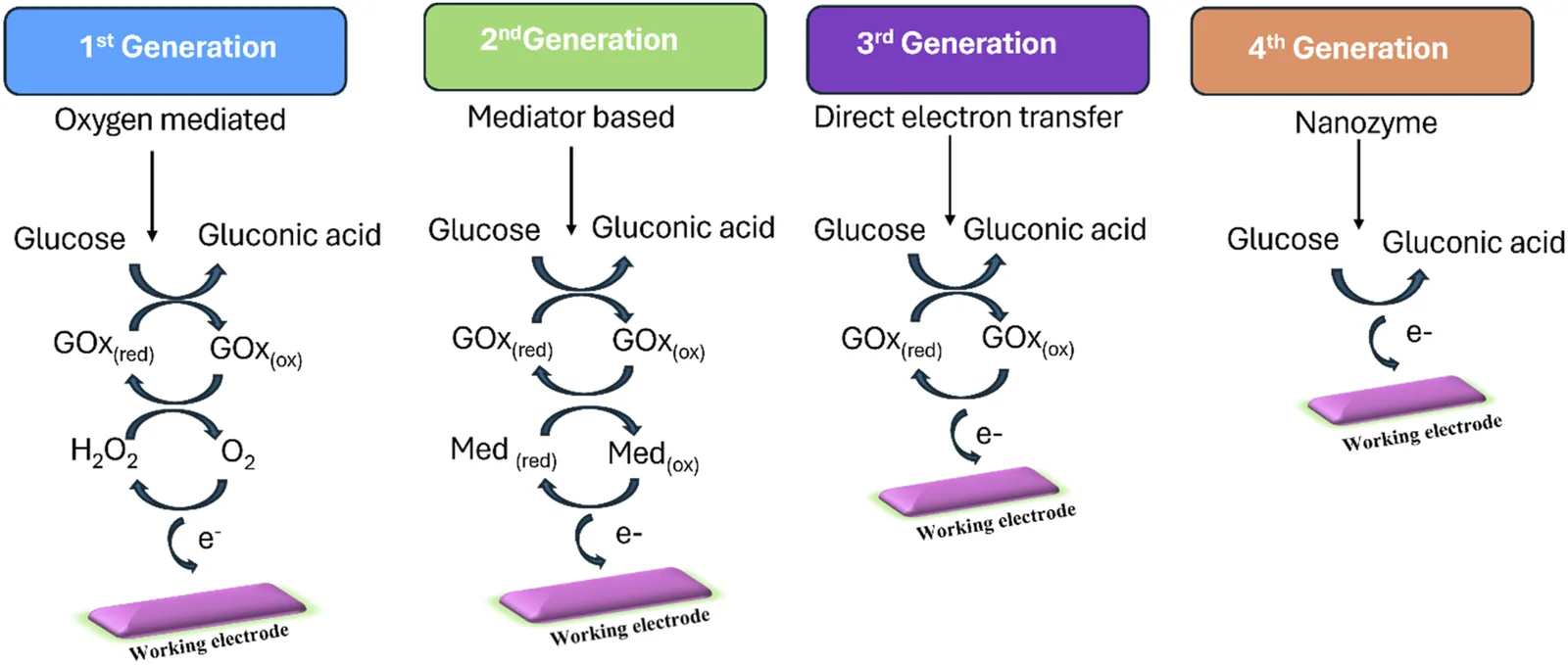
Evolution of MOF-based electrochemical glucose sensor.

### 4.1 Mechanism of MOF-based enzymatic glucose sensor

Metal–organic frameworks (MOFs) and their composites have been extensively explored as immobilisation matrices for glucose oxidase (GOx) in enzymatic electrochemical glucose sensors due to their high surface area and tunable pore structures. The abundant pores of MOFs facilitate the entrapment of GOx molecules, enabling efficient catalytic oxidation of glucose.^92,93^ Upon application of a suitable potential, GOx catalyses the oxidation of glucose to gluconic acid, generating hydrogen peroxide as a byproduct, which serves as an electroactive species for glucose quantification. A major limitation associated with pristine MOFs is the physical confinement of the relatively large GOx enzyme (6.0 × 5.2 × 7.7 nm^3^) within their pore channels.^94^ Such encapsulation can partially block pore accessibility, change the framework integrity, and ultimately compromise the structural stability and sensing performance of the materials. To replace this, functionalized MOFs containing guest accessible groups such as amino, amide, sulphonic acid and nitro functionalities have been developed.^95^ These surface functionalities enable covalent immobilisation of GOx through interaction with amine and carboxyl groups, thereby enhancing enzyme loading, stability and retention of catalytic activity. Despite these advantages, MOFs remain limited due to the prominent drawbacks of MOFs, particularly their poor electrical conductivity. The low conductivity of most pristine MOFs restricts the efficient electron transfer between the enzyme active sites and the electrode surface, thereby limiting sensor sensitivity. Consequently, considerable research efforts have focused on the development of MOF-based composites incorporating conductive metal nanostructures, carbon nanomaterials, and other electroactive components. These composites not only improve charge transport but also enhance catalytic activity and structural stability. In particular, MOF composites have been employed in biomimetic multi-enzyme sensing systems, where the immobilised GOx catalyses glucose oxidation to produce gluconic acid and H_2_O_2_.^96^ This synergistic catalytic mechanism modifies the electrochemical signal and improves glucose sensing performance.1
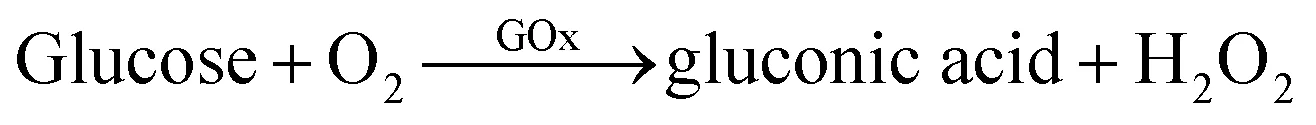
2
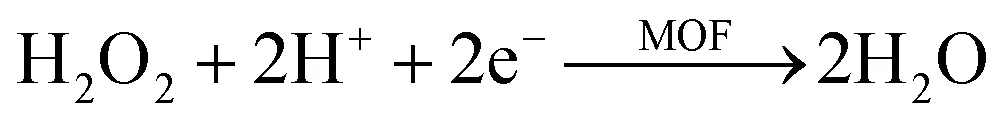


### 4.2 Mechanism of MOF-based non-enzymatic glucose sensor

The electrochemical redox properties of specific metal ions or ion clusters (M^*n*+^) within the metal–organic framework make them highly effective catalysts for various molecular redox reactions. Consequently, for efficient sensing, analytes like glucose must either be in proximity to these M^*n*+^ sites or diffuse into the MOF pores to facilitate the reaction.^97^ The glucose-sensing mechanism of MOF nanozymes generally follows a two-step process:

Step 1: glucose molecules adsorb onto the surface and migrate into the pores to reach the framework active sites.

Step 2: upon applying an electrical potential, the M^*n*+^ centres are oxidised. These centres are then reduced back to their original state through the simultaneous oxidation of glucose,^98,99^ as shown in the following reactions:

3 M^*n*+^ → M^*n*+1^ + e^−^

4 Glucose + M^*n*+1^ → gluconolactone + M^*n*+^

## 5. Enzymatic glucose sensors

Catalytic activity of biological enzymes is detected *via* an electrochemical sensor to generate electrochemical signals for target detection. The biorecognition element is a biological or synthetic molecule, such as an antibody, enzyme, nucleic acid or aptamer, which is immobilised on the surface to detect the target analyte by producing measurable signals.^100^ Enzymatic glucose sensors rely on glucose oxidase (GOx) to catalyse the oxidation of glucose to gluconolactone, producing hydrogen peroxide or consuming oxygen, which can be detected electrochemically. Conventional enzyme-based sensors suffer from challenges such as enzyme instability, poor electron transfer, and limited operational lifetime. To overcome these issues, MOFs offer several advantages for enzymatic sensing, including high enzyme loading capacity, protection against environmental stress, and controlled microenvironments that preserve enzymatic activity.^101^ The immobilisation of enzymes within the MOF depends upon how the enzyme interacts with the MOF matrix. The construction of enzyme@MOF composites can generally be divided into four main categories: physical adsorption, covalent bonding, coprecipitation (*in situ* encapsulation), and pore embedding (Fig. 3).^102–107^

**Fig. 3:**
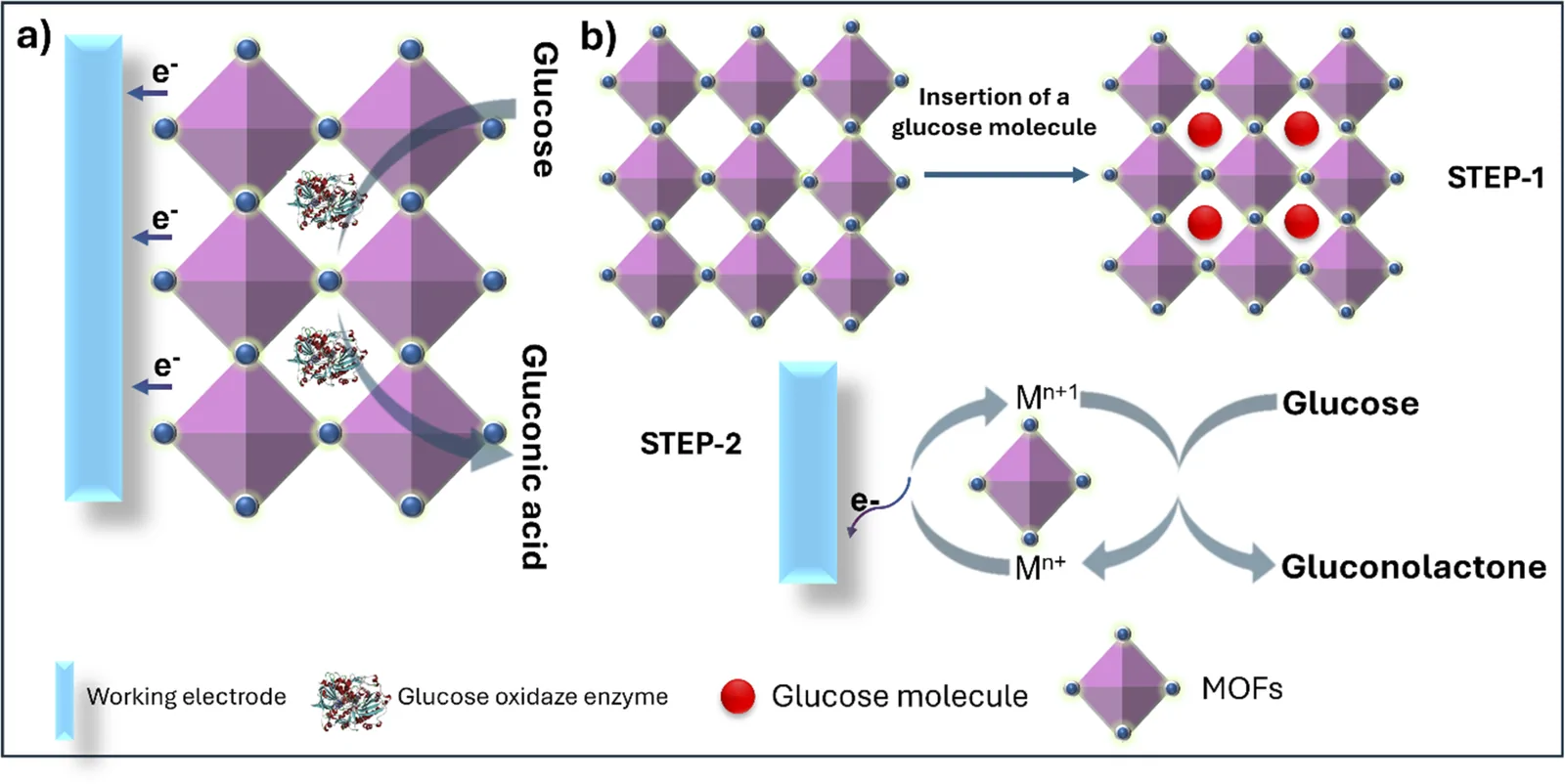
Schematic representation of MOF-based glucose sensing strategies: (a) enzymatic glucose sensor and (b) non-enzymatic glucose sensor.

### 5.1 Wearable MOF-based enzymatic glucose sensor

A wearable and flexible glucose sensor offers a noninvasive alternative to conventional blood glucose monitoring by detecting glucose in sweat, tears, and saliva. The glucose levels in these biofluids correlate with blood glucose concentration, enabling continuous monitoring through devices integrated into wearable platforms. Recent research has highlighted the potential of MOF-based materials for highly sensitive electrochemical glucose sensing in wearable applications. Wang *et al.* developed a nanocage-based zeolitic imidazole framework (NC-ZIF) network by co-encapsulating glucose oxidase (GOx) and hemin within a bimetallic Co–Zn MOF structure. The resulting GOx/Hemin@NC-ZIF electrocatalyst exhibited enhanced glucose oxidation performance and was successfully integrated into a carbon paste electrode for glucose detection. Furthermore, a wearable sweatband incorporating this sensor enabled non-invasive glucose monitoring from human perspiration, with results comparable to those obtained from conventional blood glucose monitoring systems^108^ (Fig. 4a). In another study, touch-based glucose sweat sensors have been developed using enzyme-carbon dot hybrid systems encapsulated within ZIF-8 nanostructures. In these devices, glucose oxidase (GOx) or lactate oxidase (LOx) and arginine-derived carbon dots were co-encapsulated within the MOF matrix and immobilised onto flexible Prussian blue-modified electrodes. The resulting sensors exhibited high sensitivity toward glucose and lactate detection, while maintaining excellent operational stability, thermal resistance, and reusability. Analytical results showed strong agreement with conventional assay methods^109^ (Fig. 4b). In another work, a multifunctional epidermal patch was fabricated using Hybridised Nano Porous Carbon (HNPC) derived from thermally treated core–shell ZIF-8@ZIF-67 crystals. The HNPC platform was functionalized with different sensing components to simultaneously monitor glucose, lactate, pH, and temperature. Glucose sensing was achieved by incorporating glucose oxidase and Prussian blue nanoparticles, whereas lactate detection employed lactate oxidase with a diffusion-limiting membrane. The integrated sensor demonstrated excellent electrocatalytic activity and reliable real-time sweat analysis, with improved accuracy achieved through pH and temperature compensation^110^ (Fig. 4c).

**Fig. 4:**
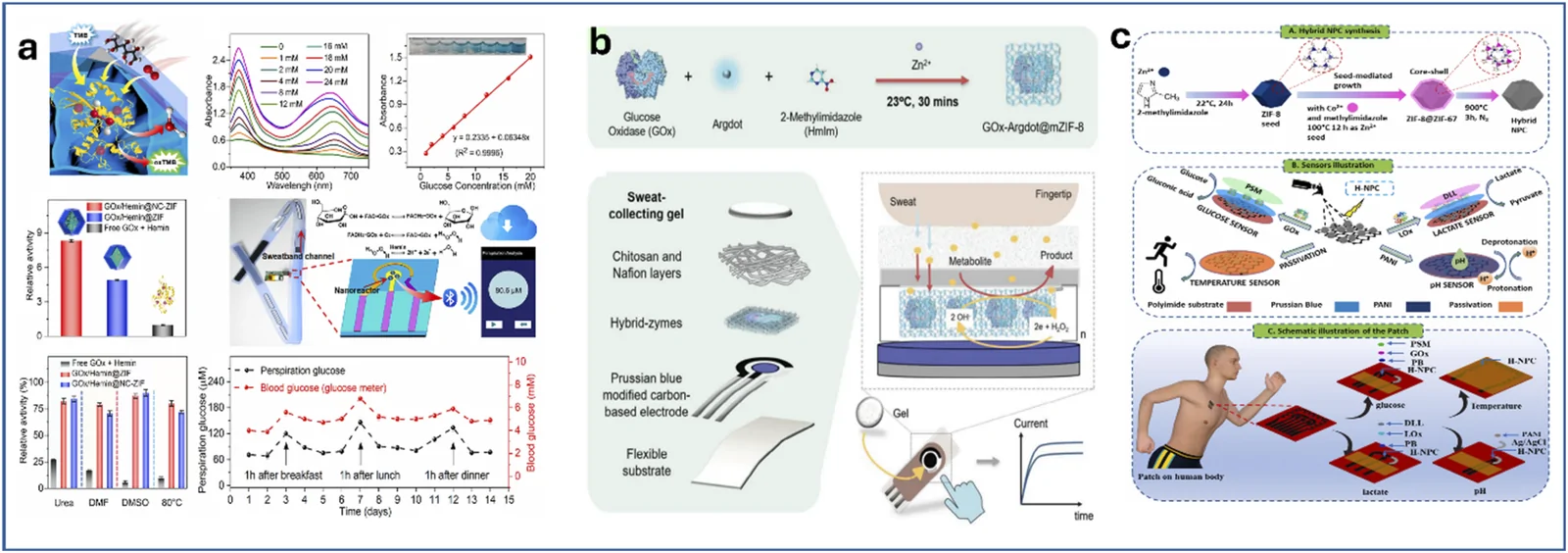
Schematic illustrations of MOF- and nanozyme-based wearable platforms for non-invasive glucose monitoring: (a) schematic illustration of the GOx/Hemin@NC-ZIF cascade nanoreactor and its application in wearable glucose sensing. Reproduced from ref. 108 with permission from Elsevier B.V.; copyright 2022. (b) Schematic illustration of the fabrication and working principle of a flexible hybrid nanozyme-based electrochemical sensor for non-invasive sweat glucose monitoring. Reproduced from ref. 109 with permission from Wiley-VCH GmbH, Weinheim, copyright 2024. (c) Schematic illustration of the fabrication of hybridised nanoporous carbon and its integration into a flexible wearable biosensing patch for simultaneous monitoring of glucose, lactate, temperature and pH. Reproduced from ref. 110 with permission from Elsevier B.V.; copyright 2023.

Overall, enzyme-MOF-based electrochemical glucose sensors significantly improve analytical performance, particularly in terms of sensitivity and detection limits, making them highly suitable for biosensing applications. However, challenges such as diffusion limitations, complex fabrication processes, and limited operational lifespan persist across these systems.^93,111^

### 5.2 Non-enzymatic glucose sensors

Non-enzymatic glucose sensors eliminate the use of fragile biological components such as glucose oxidase or glucose dehydrogenase. Instead of an enzymatic reaction, these sensors rely on the direct electrooxidation of glucose at the surface of catalytic electrodes. Transition-metal-based MOFs containing Ni, Co, or Cu centers have received significant attention because they exhibit intrinsic electrocatalytic activity for glucose oxidation in both alkaline and neutral media and their relatively low cost compared with noble metals. In particular, the redox transitions of these metals (*e.g.*, Ni^2+^/Ni^3+^or Co^2+^/Co^3+^) facilitate the oxidation of glucose into glucanolactone in alkaline or neutral electrolytes, generating measurable current signals proportional to glucose concentration. The integration of Ni, Co, or Cu based MOFs into electrode architectures significantly enhances the electrooxidation kinetics of glucose because these metal centres act as active catalytic sites, while the porous framework promotes rapid diffusion of glucose molecules to the electrode surface.^112–114^

Nickel is an earth-abundant, low-cost transition metal that has attracted significant attention for glucose sensing applications owing to its excellent electrochemical properties. The reversible Ni^2+^/Ni^3+^ redox couple plays a crucial role in facilitating glucose oxidation, enabling Ni-based MOFs to effectively exploit their abundant active sites and exhibit enhanced electrocatalytic activity. The structural and electrochemical advantages collectively contribute to improved sensitivity, lower detection limits, and faster response times, highlighting the potential of MOFs as promising platforms for high-performance glucose sensors.^115–117^ Zhang *et al.* developed a pyridine-regulated lamellar Ni-based MOF for non-enzymatic electrochemical glucose sensing. This study reports that limitations of conventional bulk MOFs include limited active site exposure and slow charge transfer. By introducing pyridine as a terminal capping ligand during hydrothermal synthesis, the authors successfully transformed bulk Ni MOFs into an ultrathin 2D lamellar structure with enhanced surface area and porosity, as seen in Fig. 5a. The optimised lamellar Ni-MOF exhibited excellent glucose sensing performance, including high sensitivity of 907.54 µA mM^−1^ cm^−2^, a wide linear detection range of 0.5–2665.5 µM, rapid response time <3 s and strong selectivity and stability. The improved sensing performance was attributed to increased active site accessibility and enhanced electron mass transfer in the 2D morphology.^118^ Further, Soni *et al.* developed a novel Ni-MOF hybrid structure composed of stacked 2D nanosheets embedded with porous nanopillars for nonenzymatic wearable glucose sensing. The hierarchical morphology provided a large electroactive surface area, abundant exposed active sites, and effective ion/electron transport pathways. The porous nanopillar network facilitated glucose diffusion and enhanced catalytic rates. Owing to these structural advantages, the Ni-MOF sensor demonstrated excellent electrochemical glucose sensing performance with a high sensitivity of 4462.03 µA mM^−1^ cm^−2^, a low detection limit of 0.28 µM. The material was successfully integrated onto flexible screen-printed electrodes, highlighting its potential for non-invasive wearable glucose monitoring using sweat and saliva samples.^119^ Likewise, Gumilar *et al.* present a generalised solvothermal strategy for synthesising hierarchical sheet/plate-like M-BDC MOFs (M–Cu, Mn, Ni and Zr) using polyvinylpyrrolidone (PVP) and acetonitrile as morphology-directing agents. The resulting architectures consist of interconnected nanosheets or nanoplates that provide enhanced surface accessibility and mass transport properties. Among the synthesised MOFs, hierarchical Ni-BDC exhibited superior non-enzymatic glucose sensing performance due to the reversible Ni^2+^/Ni^3+^ redox couple (Fig. 5b). It has a high sensitivity of 635.9 µA mM^−1^ cm^−2^, a fast response time <5 s, a low detection limit of 6.68 µM, and good selectivity without requiring conductive carbon supports. Altogether, it highlights the importance of morphology engineering in MOFs and provides an effective strategy for designing hierarchical electrocatalytic materials for biosensing applications.^120^ Similarly Wang *et al.* employed a dimensional manipulation strategy to synthesise Ni(ii) MOF with 1D, 2D, and 3D architectures and investigated the structure–activity relationship in glucose sensing (Fig. 5c and d). Among the synthesised materials, the 3D interconnected Ni-MOF (CTGU-34) exhibited superior electrochemical performance due to its well-developed porous channels, enhanced electron transport, and improved glucose diffusion kinetics. The optimised sensor achieved an ultrafast response time of less than 0.4 s, along with high sensitivity and low detection limit, demonstrating the importance of framework dimensionality and pore engineering in MOF-based electrochemical biosensors.^121^

**Fig. 5:**
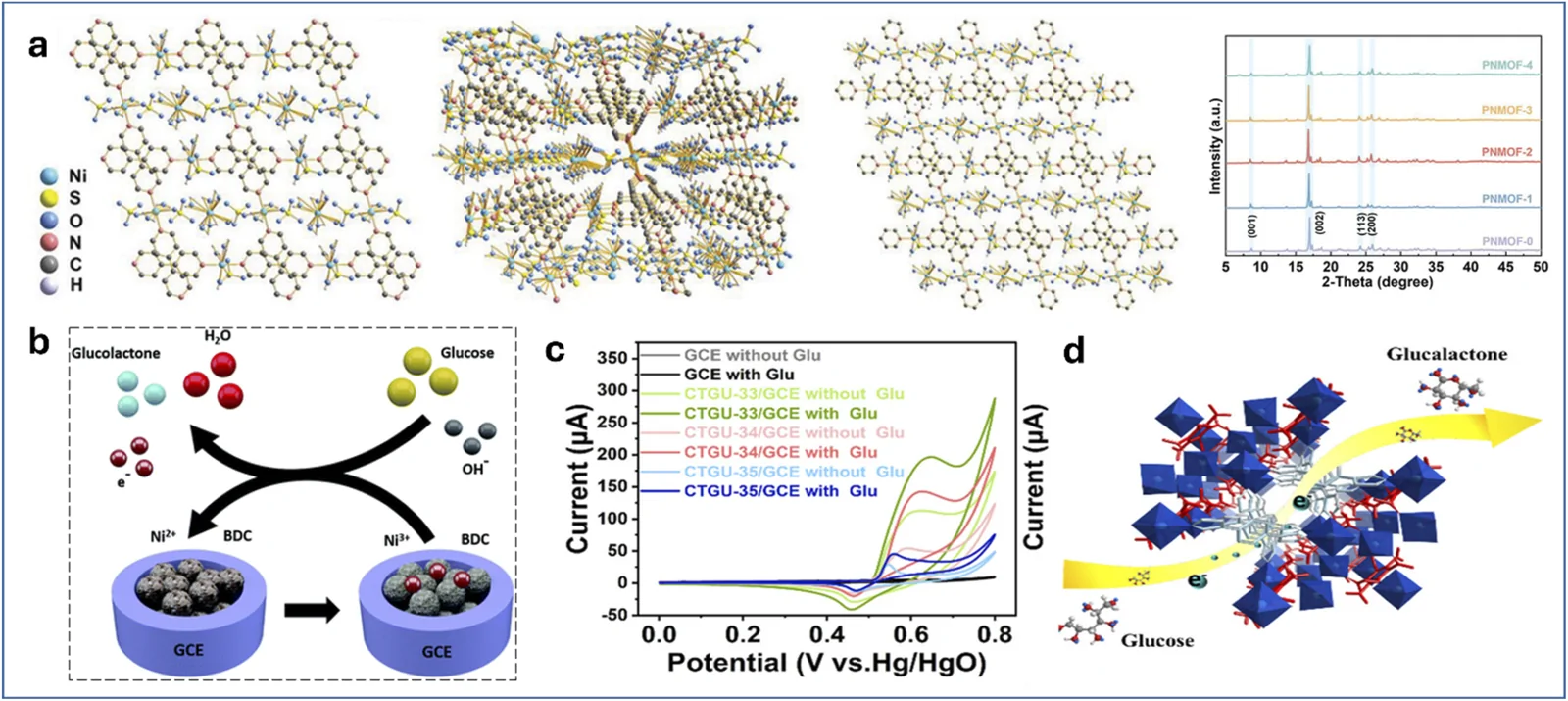
Structural and electrochemical characteristics of Ni-based MOFs for non-enzymatic glucose sensing: (a) structural analysis of the Ni-MOF series showing 2D and 3D stacking diagrams of PNMOF-0 along *a* and *c* directions, along with PXRD patterns confirming crystallinity. Reproduced from ref. 118 with permission from Wiley-VCH GmbH, Weinheim, copyright 2023. (b) Schematic illustration of the NEEGS mechanism of the Ni-BDC MOF-modified glassy carbon electrode. Reproduced from ref. 120 with permission from the Royal Society of Chemistry, copyright 2020. (c) CV curves of CTUG-33, CTUG-34, and CTUG-35 modified GCE in 0.1 M NaOH solution with and without glucose and (d) schematic illustration of the electrochemical glucose oxidation mechanism on the CTUG-34 modified electrode. Reproduced from ref. 121 with permission from the American Chemical Society, copyright 2023.

Cobalt has emerged as a promising transition metal for electrochemical glucose sensing due to its multiple accessible oxidation states and excellent properties. The reversible redox transition between Co^2+^, Co^3+^, and Co^4+^ facilitates efficient electron transfer processes, thereby enhancing the electrocatalytic activity of cobalt-based materials.^122^ Wei *et al.* developed a flexible, binder-free cobalt MOF nanosheet nanoarray grown directly on carbon cloth (Co-MOF NS/CC) for dual-function nonenzymatic glucose sensing and the oxygen evolution reaction. The electrode exhibits high glucose sensing performance with excellent selectivity, low detection limit and wide linear range. Its superior catalytic activity was attributed to a reversible Co^2+^/Co^3+^ redox-mediated glucose oxidation mechanism, enhanced electrochemical surface area, low charge transfer resistance and efficient electron transport enabled by the well-aligned 3D nanoarray architecture, as shown in Fig. 6a. Thus, the flexible carbon cloth substrate and binder-free design support its potential application in wearable and continuous glucose monitoring.^123^ Jin *et al.* investigated the structural stability and catalytic mechanism of two-dimensional cobalt-based zeolitic imidazole framework (ZIF-L) electrodes during nonenzymatic glucose oxidation. The study reveals that although ZIF-L initially exhibits good glucose-sensing performance, the material undergoes complete structural reconstruction in alkaline conditions and is transformed into CoOOH during catalysis. Through *in situ* Raman spectra, XPS and microscopic analysis, the authors confirmed that the reconstructed CoOOH phase, rather than the original MOF framework, serves as the actual active species responsible for sustained glucose oxidation, as shown in Fig. 6b. The work also exhibits a dynamic equilibrium between CoOOH and Co(OH)_2_ during the catalytic process, highlighting that MOFs primarily function as precursor materials instead of stable electrocatalysts. These studies provide key findings for the rational design of more stable and high-performance MOF-derived electrode materials for nonenzymatic and wearable glucose sensing applications.^124^ De Chiare *et al.* developed a flexible laser-induced graphene (LIG) based electrochemical sensor integrated with a ZIF-67-derived Co/Co_3_O_4_/carbon composite through a rapid UV laser scribing approach. The study highlights the limitations of conventional LIG sensors, such as low surface area, limited electrochemical activity, and poor multi-layer sensing capability, by incorporating MOF-derived porous carbon and catalytically active cobalt nanoparticles into the electrode architecture. The resulting flexible PI + Z67_L_ sensor exhibits enhanced electrochemical performance, including more than 100-fold lower impedance and 400-fold higher capacitance compared to the bare LIG electrode, due to its porous nitrogen-rich carbon matrix, uniformly distributed Co/Co_3_O_4_ core–shell nanoparticles, and efficient electron transport pathways. The sensor also demonstrated effective dopamine sensing with good selectivity, mechanical flexibility and long-term electrochemical stability, highlighting its strong potential for wearable and flexible biosensing applications.^125^

**Fig. 6:**
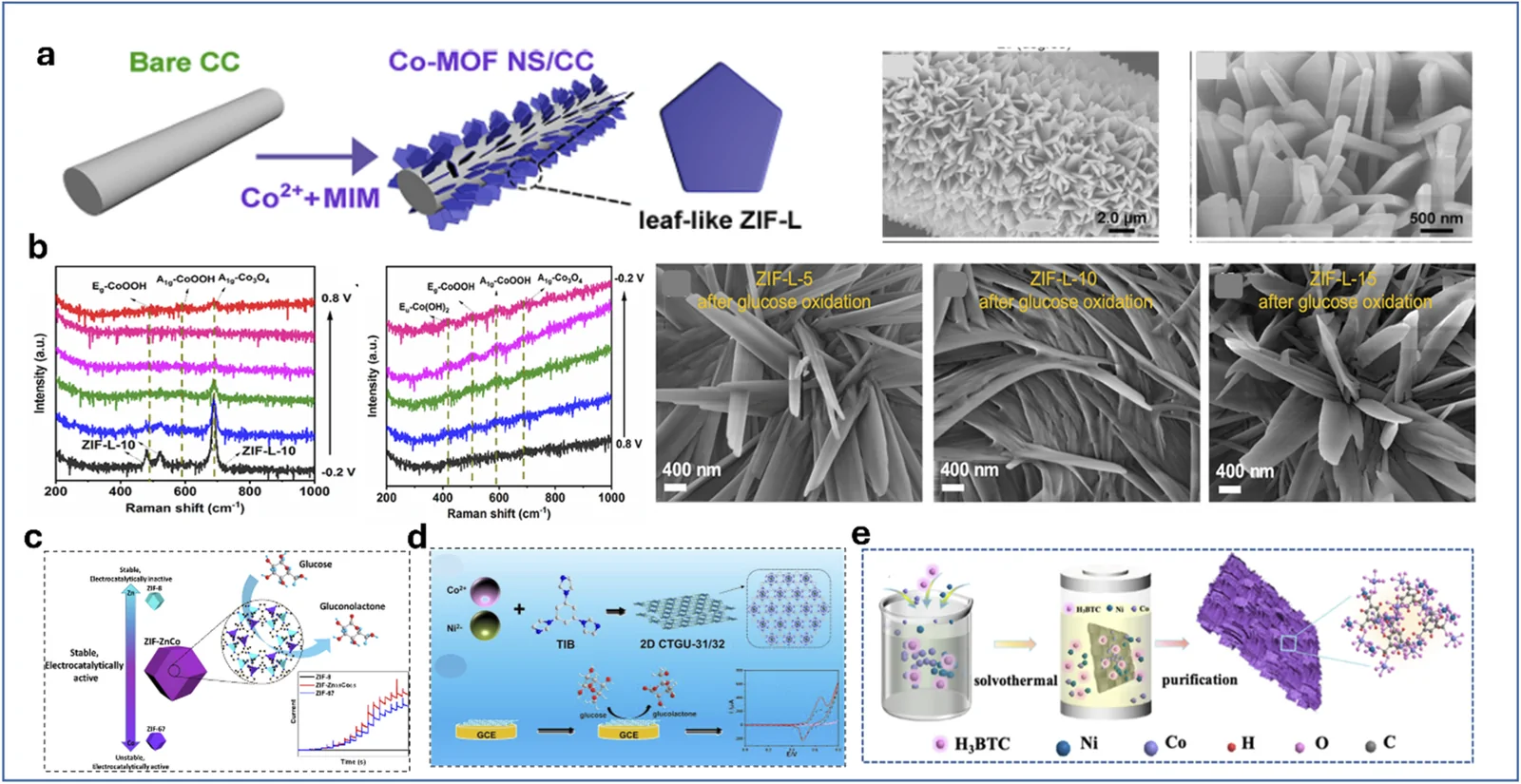
ZIF- and bimetallic MOF-based electrocatalytic platforms for non-enzymatic glucose sensing: (a) schematic illustration showing the growth of leaf-like ZIF-L nanosheets on bare carbon cloth, along with low- and high-resolution SEM images, reveals the densely packed nanosheet morphology. Reproduced from ref. 123 with permission from the American Chemical Society, copyright 2018. (b) *In situ* Raman spectra of ZIF-L-10 during oxidation and reduction processes, along with SEM images of ZIF-L-5, ZIF-L-10, and ZIF-L-15 after glucose oxidation. Reproduced from ref. 124 with permission from the American Chemical Society, copyright 2024. (c) Schematic representation of the electrocatalytic activity and chemical stability of ZIF-Zn_0.5_Co_0.5_ exhibits optimal glucose oxidation to gluconolactone with enhanced amperometric response. Reproduced from ref. 126 with permission from the American Chemical Society, copyright 2022. (d) Synthesis of 2D CTUG-31/32 *via* coordination of Co^2+^/Ni^2+^ with TIB ligand and its application as a GCE modifier for electrochemical glucose oxidation to gluconolactone with corresponding CV response. Reproduced from ref. 127 with permission from the American Chemical Society, copyright 2023. (e) Fabrication process of NiCo-BTC/CC *via* solvothermal reaction. Reproduced from ref. 128 with permission from the American Chemical Society, copyright 2022.

Among various MOF-based sensing platforms, copper-based pristine MOFs have emerged as an attractive candidate for glucose detection because of their favourable redox properties and inherent selectivity. Their low redox potential facilitates glucose oxidation at relatively low operating potentials, thereby reducing interference from electroactive species such as ascorbic acid and uric acid, as well as sugars including maltose, fructose, and sucrose. As a result, these materials exhibit enhanced analytical performance and have been widely investigated for glucose sensing applications.^129–131^ Shi *et al.* developed a Cu-based metal hydroxide organic framework containing coordinatively unsaturated copper active sites for highly efficient glucose electrooxidation. Compared with conventional Cu(OH)_2_ catalysts, the Cu-MHOF exhibited nearly 40- fold higher electrocatalytic activity and enabled the complete oxidation of glucose into formate and carbonate. The enhanced catalytic performance was attributed to the open copper active sites, which improved the adsorption of glucose and reaction intermediates, as supported by control experiments and density functional theory simulations. It shows a high sensitivity of 214.7 µA mM^−1^ cm^−2^, a wide linear detection range of 0.1 µM to 22 mM, and a low detection limit of 0.086 µM. This study highlights a molecule-level strategy for designing highly active MOF-based electrocatalysts for advanced electrochemical sensing applications.^132^ In addition to this, Li *et al.* developed porous crystalline CuO architectures derived from Cu-BTC MOFs for a non-enzymatic glucose sensor. The CuO structures were synthesised through thermal annealing and characterised using XRD, SEM, TEM and XPS, confirming their porous and crystalline nature. The porous morphology enhanced the electrocatalytic surface area and glucose diffusion, resulting in excellent electrocatalytic activity. The fabricated CuO sensor exhibits a high sensitivity of 934.2 µA mM^−1^ cm^−2^, a low detection limit of 0.1 µM, a rapid response time of 1.3 s, and a wide linear range from 0.5 µM to 2.8 mM. Additionally, the sensor showed excellent selectivity, reproducibility, and long-term stability for glucose detection.^133^ Similarly, Xie *et al.* developed a non-enzymatic electrochemical glucose sensor using Cu MOF (HKUST-1) derived Cu nanospheres embedded in porous carbon supported on three-dimensional kenaf stem-derived macroporous carbon (3D-KSCs). The hierarchical porous structure enhanced electron transfer, catalytic activity, and glucose oxidation efficiency. The sensor exhibits high sensitivity of 28.67µA mM^−1^ cm^−2^, a low detection limit of 4.8 µM, and has a rapid response and excellent selectivity against interfering biomolecules. The work addressed major limitations of enzyme-based and conventional Cu-based sensors, including poor stability, nanoparticle aggregation, and low conductivity, demonstrating a low-cost and stable platform for electrochemical glucose sensing applications.^134^

Furthermore, bimetallic or trimetallic MOF systems containing combinations of Ni, Co and Cu often exhibit synergistic catalytic effects, resulting in improved sensitivity, lower detection limits, and wider linear detection ranges in contrast to single-metal catalysts. These enhanced performances are attributed to improved electrical conductivity, increased active surface area, and optimised adsorption of glucose within the porous structure.^135,136^ Kim *et al.* reported Zn–Co bimetallic zeolitic imidazolate framework (ZIF-Zn_*x*_Co_1−*x*_) as chemically stable nonenzymatic glucose sensing materials. By partially substituting cobalt with zinc, they improved the alkaline stability of Cobalt-based ZIFs while preserving their electrocatalytic activity. Among the synthesised compositions, ZIF-Zn_0.5_Co_0.5_ exhibits optimal performance, retaining its crystallinity and porosity after prolonged exposure to alkaline media. Electrochemical studies revealed high glucose sensing sensitivity of 1105.6 µA mM^−1^ cm^−2^, a low detection limit of 9 µM and excellent selectivity against common interfering species. The study highlights the potential of bimetallic MOF engineering for developing high-performance nonenzymatic electrochemical biosensors^126^ as shown in Fig. 6c. Similarly Wang *et al.* developed two-dimensional isomorphic Co/Ni MOFs, namely CTGU-31 and CTGU-32, for a nonenzymatic glucose sensor. The nitrogen-rich ligand 1,3,5-tris(1-imidazolyl)benzene (TIB) produces layered MOF structures with abundant electroactive sites and high surface area. To improve its conductivity, the MOFs were integrated with conductive acetylene black (AB), and the optimised AB/CTUG-32 (1 : 1) composite exhibits superior sensing characteristics, as shown in Fig. 6d. The enhanced sensing performance was attributed to the synergistic combination of the conductive acetylene black and the unique two-dimensional Ni-MOF layered structure, highlighting the potential of conductive MOF composites for high-performance non-enzymatic biosensing applications.^127^ Further, Zha *et al.* developed an ultrathin bimetallic NiCo-BTC MOF nanosheet array on Carbon Cloth (NiCo-BTC/CC) for non-invasive electrochemical glucose sensing. The morphology-controlled ultrathin nanosheet structure provided a high surface area, enhanced active site exposure, and improved electron transport, leading to superior electrocatalytic activity, as shown in Fig. 6e. The sensor shows good stability, reproducibility, and successful noninvasive glucose detection in sweat, showing results that are comparable to commercial invasive glucose testing devices.^128^ This study shows the bimetallic design for developing a highly sensitive wearable glucose sensor. Finally, compared with enzymatic sensors, MOF-based non-enzymatic systems offer several advantages, including greater chemical stability, longer operational lifetime, resistance to environmental conditions such as pH and temperature variations and simpler fabrication processes. Despite these advantages, challenges remain in achieving high selectivity in complex biological matrices because interfering species such as ascorbic acid, uric acid, and dopamine may undergo oxidation at similar potentials. These systems offer improved stability and simplified fabrication compared to enzymatic sensors, although achieving high selectivity under physiological conditions remains challenging.

### 5.3 Strategies for enhancing the electrical conductivity of MOFs

Despite their remarkable surface area, tunable porosity and abundant chemically active sites, many conventional MOFs exhibit intrinsically poor electrical conductivity, which limits their direct application in electrochemical sensing. This low conductivity is primarily associated with localised electronic states and weak electronic coupling between metal nodes and organic linkers, resulting in inefficient charge transport, high charge transfer resistance, and sluggish electron-transport kinetics. To overcome this limitation, several conductivity enhancement strategies have been developed.^84,126^ These approaches can be broadly classified into three categories: (1) integration of MOFs with extrinsic conductive materials, which includes carbon materials, metal nanoparticles, and conductive polymers; (2) construction of intrinsically conductive MOFs; and (3) pyrolysis or carbonisation of MOFs to generate conductive carbon-based derivatives. Importantly, these strategies do not provide equivalent benefits: conductive composites generally preserve the parent MOF structure, intrinsically conductive MOFs provide framework-level charge transport, whereas MOF-derived carbons achieve high conductivity at the expense of substantial transformation of the original framework.

#### 5.3.1 MOF-conductive material composites

Integration of MOFs with extrinsic conductive materials represents the most widely adopted approach for overcoming the poor electrical conductivity of conventional MOFs. In these hybrid systems, the conductive component establishes additional electron-transport pathways, while the MOF retains its porous architecture and provides accessible catalytic, adsorption, or redox-active sites. Conductive carbon materials, particularly graphene, reduced graphene oxide (rGO), carbon nanotubes (CNTs), and carbon nanofibers (CNFs), are attractive because of their high electrical conductivity, large surface area, and ability to form interconnected conductive networks.^137,138^

Graphene and rGO can provide two-dimensional electron-delocalisation pathways and close interfacial contact with MOF crystallites. Nagarajan *et al.* reported a base-free Cu-MOF/rGO composite for glucose sensing and energy storage applications, in which rGO enhanced charge transport, surface accessibility, and structural stability, while Cu–N_4_ sites provided the electroactive centres for glucose oxidation. The resulting composite exhibited a sensitivity of 4036 µA mM^−1^ cm^−2^ and a detection limit of 0.4 µM, demonstrating the synergistic contribution of the conductive carbon phase and MOF active sites. The reported result indicates that the incorporation of rGO can improve electron mobility without requiring complete transformation of the MOF framework, as shown in Fig. 7a.^139^

**Fig. 7:**
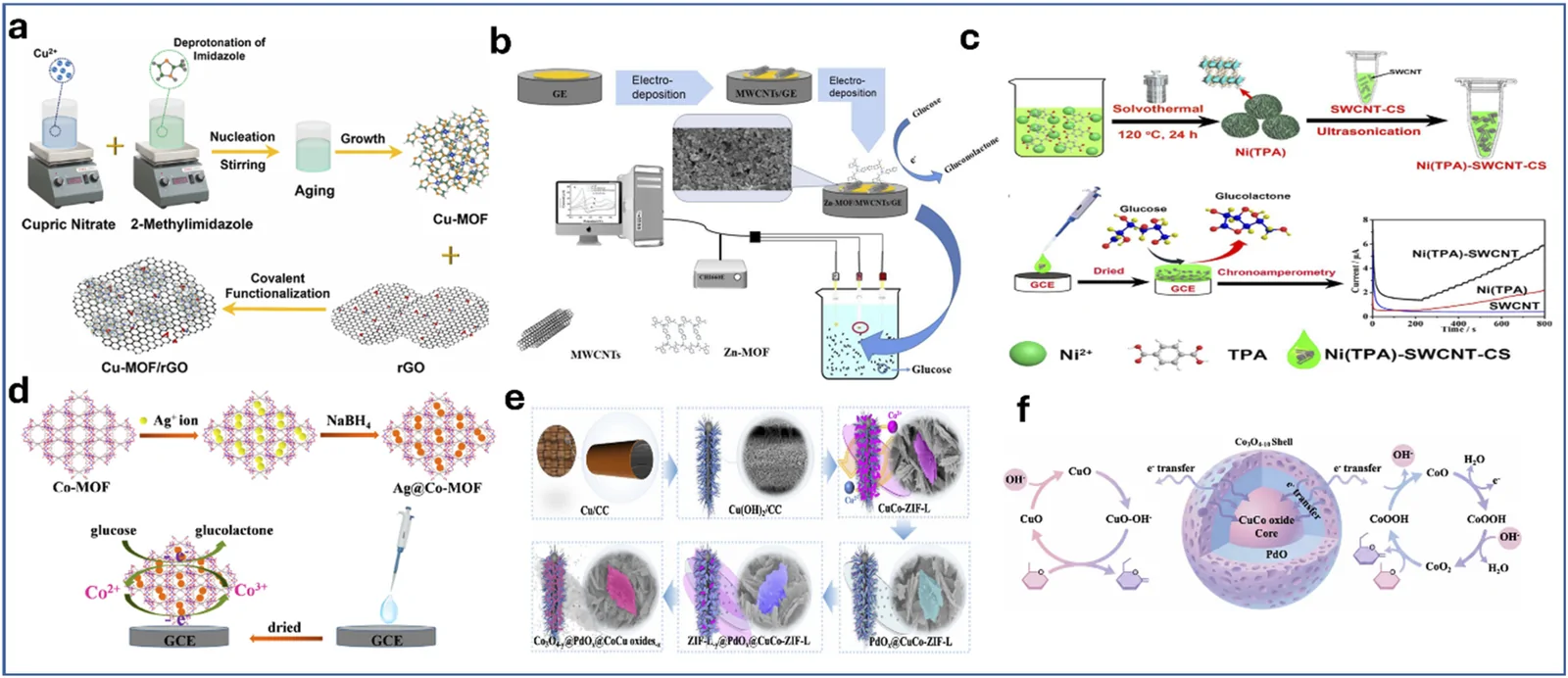
(a) Synthesis route of Cu-MOF/rGO composites *via* covalent functionalization with rGO. Reproduced from ref. 139 with permission from the American Chemical Society, copyright 2025. (b) Fabrication of Zn-MOF/MWCNTs/GCE *via* sequential electrodeposition of MWCNTs and Zn-MOF for a non-enzymatic electrochemical glucose sensor. Reproduced from ref. 140 with permission from Elsevier B.V., copyright 2024. (c) Synthesis of Ni(TPA)/SWCNT composite *via* the solvothermal method and its application. Reproduced from ref. 141 with permission from Elsevier B.V., copyright 2019. (d) Preparation of Ag@Co-MOF followed by drop casting on GCE. Reproduced from ref. 142 with permission from the American Chemical Society, copyright 2019. (e) Stepwise fabrication process from Cu/CC to Co_3_O_4−*y*_@PdO_*x*_@Cu oxides_−400_*via* sequential transformation through Cu(OH)_2_/CC, CuCo-ZIF-L, PdO_*x*_@CuCo-ZIF-L and ZIF-L_−*y*_@CuCo-ZIF-L intermediate and (f) an electrochemical glucose sensing mechanism showing electron transfer through the core–shell structures. Reproduced from ref. 143 with permission from Royal Society of Chemistry, copyright 2026.

One-dimensional carbon materials, such as CNTs and CNFs, can serve as conductive bridges between MOF crystallites, facilitating electron permeation through the electrode. Xue *et al.* developed an enzyme-free Zn-MOF/MWCNT glucose sensor, as shown in Fig. 7b, where the MWCNT network increases the electrochemically accessible surface area and facilitates electron transfer, enabling glucose oxidation at a relatively low potential of 0.20 V. The sensor exhibited a linear range of 0.020–8.14 mM and a detection limit of 0.037 mM, together with good reproducibility and successful application to human serum samples.^140^ Similarly, Dey *et al.* developed a flexible, binder-free Ni(PDA)MOF@CNF electrode through an *in situ* solvothermal process. The conductive CNF network was integrated with redox-active Ni-MOF, producing a porous hybrid architecture with efficient charge transport and abundant accessible catalytic sites. The sensor exhibited a sensitivity of 9457.5 µA mM^−1^ cm^−2^, a detection limit of 0.053 µM, a linear range of 10–3000 µM, and a response time of approximately 2 s.^144^ Similarly, Wang *et al.* constructed a hierarchical three-dimensional flower-like Ni(TPA)MOF/SWCNT/chitosan electrode. The porous Ni(TPA) architecture supplied abundant Ni^2+^/Ni^3+^ active centres, while SWCNTs increased the electroactive centres by approximately 39-fold, and the resulting electrode exhibited a linear glucose response from 20 µM to 4.4 mM, a detection limit of 4.6 µM, and a response time below 5 s, as shown in Fig. 7c.^141^

Metal nanoparticles provide another effective route for improving charge transport because they combine high electrical conductivity with catalytic functionality. Chen *et al.* developed Au nanoparticles deposited on Ni-BTC microspheres as an efficient non-enzymatic glucose oxidation through synergistic interactions between conductive Au nanoparticles and Ni^2+^/Ni^3+^ redox centres, resulting in a sensitivity of 1447.1 µA mM^−1^ cm^−2^ and a detection limit of 1.5 µM.^142^ Similarly, Liu *et al.* report the development of a multifunctional Ag nanoparticle-encapsulated cobalt MOF (Ag@Co-MOF) for non-enzymatic glucose sensing and magnetic applications. A porous 3D Co-MOF containing pentanuclear Co(ii) clusters was synthesised using 5,5′-oxidiisophthalic acid as the organic linker. To improve the poor conductivity of the MOF, silver nanoparticles were incorporated into the porous framework through a deposition–reduction method. Structural analyses confirmed the successful and uniform distribution of Ag nanoparticles within the MOF pores without destroying the framework, as shown in Fig. 7d. The Ag@Co-MOF modified electrode exhibits enhanced glucose oxidation performance with a high sensitivity of 0.135 µA µM^−1^, a low detection limit of 1.32 µM, excellent selectivity and good operational stability.^145^ Further, Meng *et al.* revealed the development of a novel Ag@ZIF-67 nanocomposite for non-enzymatic glucose sensing. ZIF-67, a cobalt-based MOF, was directly utilised as an electrocatalyst for glucose oxidation, while silver nanoparticles were incorporated into its porous framework through a sequential deposition–reduction method to improve conductivity and catalytic activity. Structural analyses confirmed the successful encapsulation and uniform distribution of Ag nanoparticles without damaging the MOF structure. The improved performance was attributed to the synergistic combination of the porous MOF and the high conductivity of Ag nanoparticles, which accelerates electron transfer and increases accessible active sites. The optimised Ag-0.5%@ZIF-67 electrode shows a wide linear detection range of 2–1000 µM, high sensitivity of 0.379 µA µM^−1^ cm^−2^ and a low detection limit of 0.66 µM, fast response time and excellent electrocatalytic activity for reliable glucose detection applications.^146^

Thus, MOF-conductive material composites provide a relatively facile and versatile means of enhancing electrical conductivity while preserving a substantial fraction of the parent MOF structure. Carbon materials primarily establish conductive percolation networks, whereas metal nanoparticles can additionally introduce catalytic active sites. Nevertheless, the resulting electrochemical performance is highly dependent on the conductive-component loading, particle dispersion, interfacial contact, and electrode fabrication procedure. Excessive incorporation of conductive additives may block MOF pores or shield active sites, whereas insufficient loading may fail to establish an effective conductive network. Therefore, optimisation of the conductive-phase content is essential for achieving a balance between conductivity and preservation of MOF functionality.

#### 5.3.2 Intrinsically conductive MOFs

A fundamentally different strategy is the development of intrinsically conductive MOFs (cMOFs), in which charge transport originates from the electronic structure of the coordination framework itself rather than from an externally introduced conductive phase. In these systems, extended π–π conjugation, strong metal–ligand orbital overlap, redox-active metal centres, and efficient charge delocalisation can generate continuous electronic pathways through the framework. A landmark example is Ni_3_(HITP)_2_, reported by Sheberla *et al.*, which exhibited conductivities of approximately 2 S cm^−1^ in pressed pellets and 40 S cm^−1^ in thin films, demonstrating that porous coordination frameworks can simultaneously exhibit high surface area and substantial electrical conductivity.^147^ Subsequent studies have expanded the application of conjugated conductive MOFs in electrochemical sensing. An electrochemically activated Ni_3_(HITP)_2_ electrode was demonstrated for non-enzymatic glucose detection over 0–10 mM, with good selectivity and stability, illustrating the direct use of a conductive MOF without requiring a conventional conductive additive.^48^ Similarly, Ni_3_(HITP)_2_ has also been incorporated into molecularly imprinted electrochemical biosensors for direct glucose detection in whole blood, achieving a detection limit of 0.31 µM over a broad concentration range.^148^ Further, Ko *et al.* employed two-dimensional Ni- and Cu based HHTP/HITP conductive MOFs as drop-cast electrodes for voltammetric detection of dopamine and serotonin, demonstrating that conductive MOFs can directly function as electrochemical transduction layers without requiring conventional carbon conductive additives.^149^ Another recent study has demonstrated electrochemical growth of Cu_3_(HHTP)_2_ directly from Cu nanoparticles, providing uniform MOF films suitable for chemiresistive sensing. This strategy offers improved control over MOF distribution and electrode integration compared with conventional post-deposition approaches.^150^ Therefore, intrinsically conductive MOFs provide a fundamentally different solution to the conductivity problem. Their major advantage is that the conductive pathway is integrated directly into the framework, eliminating phase segregation and reducing dependence on the dispersion of an external conductive additive. They can also preserve molecularly defined porosity while providing electronic transport. However, the conductivity of cMOFs is strongly affected by crystallinity, defects, stacking configuration, orientation, film thickness, and electrode-MOF contact. Moreover, synthesis of highly crystalline conductive frameworks and reproducible integration onto practical electrode substrates can be more demanding than conventional composite fabrication. Thus, while cMOFs offer superior conceptual control over charge transport, their scalability and reproducibility remain important considerations.

#### 5.3.3 MOF-derived conductive carbon materials

A third strategy involves the thermal conversion or pyrolysis of MOFs into conductive carbon-based materials. During carbonisation, the organic ligands are transformed into carbonaceous frameworks, while metal centres can generate metallic oxides, carbides, or other catalytically active nanoparticles. The resulting materials generally exhibit enhanced electrical conductivity, hierarchical porosity, improved mechanical stability, and abundant electrochemically accessible active sites.^151^ However, unlike conductive MOF composites, pyrolysis fundamentally transforms the parent MOF structure and therefore sacrifices part or all of its original crystallinity, coordination environment and molecularly defined pore chemistry. ZIF-derived carbon materials are particularly attractive because the homogeneous distribution of metal centres within the precursor MOF can be converted into highly dispersed catalytic nanoparticles embedded in a porous carbon matrix. ZIF-derived carbon materials are particularly attractive because the homogeneous distribution of metal centres within the precursor MOF can be converted into highly dispersed catalytic nanoparticles embedded in a porous carbon matrix. Shi *et al.* reported ZIF-67-derived porous carbon for glucose oxidase immobilisation and glucose sensing. During carbonisation, Co nanoparticles catalysed carbon graphitisation, improving electrical conductivity, while subsequent acid etching generated additional pores and increased the accessible surface area. The resulting porous carbon facilitated enzyme immobilisation and rapid electron transfer. The electrochemical properties of MOF-derived carbon materials are strongly influenced by the carbonisation atmosphere and temperature.^152^ Bimetallic MOF precursors can further introduce synergistic catalytic effects. For example, MnCo-MOF-74-derived Co/MnO@hierachical carbon was prepared by high-temperature carbonisation and subsequently used for non-enzymatic glucose sensing. The resulting material exhibited a sensitivity of 233.8 µA mM^−1^ cm^−2^ and a detection limit of 1.31 µM over two linear concentration ranges, demonstrating the combined advantage of a conductive hierarchical carbon matrix and bimetallic catalytic centres.^153^ Further, heteroatom-doped MOF-derived carbons provide an additional route for modifying electronic properties. Nitrogen-containing carbon frameworks can introduce pyridinic, pyrrolic, and graphitic nitrogen sites that influence charge transport, surface polarity, and catalytic activity. Yan *et al.* developed Co/CoO nanoparticles embedded in n-doped nanoporous carbon derived from ZIF-67. The resulting material exhibited a sensitivity of 143.9 µA mM^−1^ cm^−2^, a wide linear range of 0.01–16.65 mM, and a detection limit of 0.8 µM for glucose sensing.^154^ MOF-derived conductive carbons offer an effective strategy for improving electron transport while generating porous architectures. Xu *et al.* converted ZIF-8 into three-dimensional nitrogen-doped porous carbon (NCZIF-8) through carbonisation at 900 °C and subsequently decorated it with 10 wt% Pt nanoparticles. The resulting material retained the original polyhedral morphology while exhibiting a high surface area of 944 m^2^ g^−1^ and hierarchical micro/mesoporosity. The porous N-doped carbon effectively dispersed Pt nanoparticles, reducing their average size to 2.7 ± 0.3 nm and increasing the electrochemically active surface area to 0.42 cm^2^. The Pt/NCZIF-8 electrode showed a low charge-transfer resistance of 2.3 Ω, indicating enhanced electron-transfer kinetics. Glucose oxidation occurred mainly at Pt sites through glucose adsorption, C–H bond cleavage, hydroxyl-mediated oxidation, and formation of gluconolactone. The electrode showed improved stability during 300 cycles and delivered a maximum glucose-fuel-cell power density of 0.54 µW cm^−2^. However, conventional sensitivity and LOD were not reported, and pyrolysis transformed the original ZIF-8 structure.^155^

In addition, MOF-derived heterostructures can combine conductive carbon, metal/metal-oxide phases, and spatial confinement. In the same way, the development of a MOF-derived sandwich heterostructure electrode, Co_3_O_4−10_@PdO_5_@CoCu oxides, enabled highly sensitive and stable non-enzymatic glucose sensing. The sensor was fabricated by incorporating PdO nanoparticles into CuCo-ZIF-L derived metal oxides, followed by the construction of a sandwich architecture using an additional ZIF-L shell. The MOF-derived porous structure provided abundant active sites and enhanced mass transport, while the incorporation of PdO significantly improved electron-transfer kinetics through synergistic interactions among CuO, Cu_3_O_4_, and PdO. The sandwich heterostructures effectively suppress PdO aggregation and prevent leaching during electrochemical cycling, thereby enhancing long-term stability, as shown in Fig. 7e and f. The optimised electrode exhibits excellent sensing performance with sensitivities of 4.372 mA mM^−1^ cm^−2^ and 2.615 mA mM^−1^ cm^−2^ and a rapid response time of 2.35 s. In addition, the sensor showed strong anti-interference capability against common biomolecules and retained 93.73% of its initial current response after 30 days. The enhanced performance was attributed to the synergistic trimetallic oxide interactions, increased exposure of active sites after annealing, and the protective role of the sandwich MOF shell.^143^ Similarly, the authors synthesised the Fe_3_O_4_@Au@CoFe-LDH structure through a spontaneous galvanic displacement reaction. In this design, metallic gold is confined between Fe_3_O_4_ nanoparticles and cobalt–iron layered double hydroxide, forming a unique sandwich architecture. The LDH acts as both a substrate and prevents gold aggregation. Electrochemical studies revealed that Au acts as the active catalytic site for glucose oxidation, where confined Au forms Au–OH active species under alkaline conditions, which catalyse glucose oxidation into gluconate. While Fe_3_O_4_ and LDH improve structural stability and electron transfer. The optimised sensor exhibits excellent sensing performance with a high sensitivity of 6342 µA mM^−1^ cm^−2^, a wide linear detection range of 0.0375–15.64 mM, a low detection limit of 12.7 µM, and a low oxidation potential of 0.82 V *vs.* RHE. The sensor also demonstrated excellent selectivity and long-term stability.^156^

Thus, MOF-derived conductive carbons offer excellent electrical conductivity, hierarchical porosity, catalytic activity, and structural robustness. Nevertheless, these benefits are obtained at the expense of transformation of the parent MOF architecture. In addition, variations in precursor composition and pyrolysis conditions can substantially modify the degree of graphitisation, pore structure, metal-species distribution, and electrochemical activity. Consequently, precise control of the carbonisation process is essential for achieving reproducible material properties.

#### 5.3.4 Critical comparison of conductivity-enhancement strategies for MOFs

**Table:** 

Performance parameter	MOF-conductive material composites	Intrinsically conductive MOFs (cMOFs)	MOF-derived conductive carbons
Basic strategy	Integration of MOFs with conductive carbon materials, metal nanoparticles, or conducting polymers	Electrical conductivity is incorporated within the MOF framework through extended electronic conjugation and charge transport pathways	Pyrolysis/carbonization of MOFs to generate conductive carbon frameworks with dispersed metal/metal oxide/metal carbide species
Structural preservation	Parent MOF structure can largely be retained	The conductive framework itself remains the sensing structure	Pyrolysis substantially transforms or destroys the original coordination framework
Sensitivity	Generally high due to combined MOF adsorption and conductive pathways; strongly dependent on composition and loading	Potentially high because molecularly defined pores and intrinsic conductivity can facilitate analyte transport and charge transfer	Often high because of high conductivity, hierarchical porosity, and abundant catalytic sites
Reproducibility and fabrication	Affected by conductive-phase loading, dispersion, and electrode preparation	Affected by crystallinity, grain boundaries, film thickness, and electrode integration	Strongly dependent on pyrolysis temperature, atmosphere, heating rate, and duration

The comparison demonstrates that no single conductivity-enhancement strategy is universally superior. MOF-conductive material composites provide a practical balance between enhanced electron transfer, preservation of MOF porosity, flexibility, and facile electrode fabrication, making them particularly attractive for wearable electrochemical sensors. Intrinsically conductive MOFs offer a more fundamental solution by incorporating charge-transport pathways within the framework itself, although their performance can be sensitive to crystallinity, grain boundaries, crystal orientation, and electrode integration. In contrast, MOF-derived conductive carbons provide high electrical conductivity, abundant accessible catalytic sites, hierarchical porosity, and excellent mechanical and chemical robustness, but at the cost of substantial transformation or loss of the parent MOF structure. Therefore, the selection of a conductivity-enhancement strategy should be application-specific and should consider not only sensitivity and detection limit but also charge-transfer resistance, active-site accessibility, structural preservation, reproducibility, fabrication complexity, flexibility, and long-term stability.

## 6. Integration of MOFs into wearable electrochemical devices

Metal–organic frameworks (MOFs) are increasingly used in wearable electrochemical sensors because they offer high precision and rapid results. Significant progress has been made, as (Table 1) Several critical challenges still need to be addressed in the near future to advance the applicability of MOF-based wearable sensors. While currently most wearable sensors only track physical activity and vital signs, the MOFs show great potential for advanced medical applications, including disease diagnosis and monitoring.^157^ However, several challenges must be overcome to fully integrate them into health care.^158,159^ A primary obstacle is the reliance on blood samples, which remains an invasive process with risks of infection and storage difficulties.^160^ To address this, current research is shifting towards the development of sensors that can continuously and non-invasively analyse biofluids such as sweat, tears, and saliva.^161^

Most recent advances in MOF-based wearable sensors and related limitations

**Table:** 

Strength	Weaknesses
➢ High porosity and surface area	➢ High fabrication cost
➢ Tunable structure	➢ Stability concerns
➢ Easy fabrication	➢ Calibration challenges
➢ High flexibility and stretchability	➢ Potential toxicity

**Table:** 

Opportunities	Threats
➢ AI-integrated wearable diagnosis	➢ Competition from nanomaterial sensors
➢ Green and biodegradable MOFs	➢ Regulatory approval barriers
➢ Wireless health monitoring systems	➢ Commercialisation challenges
➢ Self-powered wearable platforms	➢ Long-term reliability concerns

Although MOF-based wearable sensors demonstrate promising laboratory-scale performance, their translation into clinical devices remains challenging. Commercial continuous glucose monitoring (CGM) systems benefit from established manufacturing, calibration, clinical validation, long-term stability and regulatory frameworks.^162^ In contrast, most MOF-based glucose sensors remain at the prototype stage and require validation under realistic physiological conditions. Variation in sweat composition, pH, ionic strength, sweat rate, temperature, and interfering biomolecules can affect sensor performance; therefore, accuracy, selectivity, reproducibility, and correlation with clinically relevant glucose levels must be demonstrated using human samples.^163^ Specific examples illustrate both the potential and translational challenges of non-invasive and sweat-based glucose monitoring. The GlucoWatch G2 Biographer, one of the earliest commercial non-invasive glucose monitoring devices, received FDA approval in 2001, demonstrating the feasibility of regulatory translation but also highlighting the challenges associated with achieving reliable long-term performance.^164^ SugarBEAT represents another approach based on non-invasive transdermal glucose extraction and has received CE marking, although further clinical validation and regulatory approval are required for broad adoption.^165^ More recent research platforms, including textile-based sweat glucose sensors with pH compensation developed at the University of Texas at Dallas and multifunctional wearable patches reported by Seoul National University, further demonstrate advances toward clinically relevant sweat-based monitoring.^166,167^ However, fully commercialised, FDA-approved sweat-based continuous glucose monitoring remains unavailable, emphasising the need for improved accuracy, physiological correlation, calibration, long-term stability, biocompatibility, and scalable manufacturing. Beyond glucose, wearable sweat sensors have demonstrated potential for monitoring lactate and uric acid associated with exercise and oxidative stress, as well as cortisol for stress-related monitoring and other clinically relevant analytes such as nicotine and levodopa. These developments highlight the versatility of advanced materials and electrochemical technologies for non-invasive health monitoring. Nevertheless, successful clinical adoption will require rigorous human-sample validation, standardised performance assessment, regulatory evaluation, and cost-effective manufacturing. Further developments integrating electrochemical and optical sensing, miniaturised electronics, and wireless platforms could further expand the applications of wearable sweat sensors in diabetes management, sports monitoring, and neurological and stress-related healthcare.^168^

### 6.1 Flexible substrates and electrode design

Today, wearable device fabrication and design are largely guided by the fields of electrochemistry, organic electronics, solar cells and large-area flexible electronics. These devices commonly use all-solid-state ion-selective electrodes, polymers, and membranes, with a focus on low cost, miniaturisation, reliability, minimal sample volume, and simple design.^169–171^ The choice of sensing materials and techniques depends on the target sweat biomarker, particularly its molecular properties and concentration. Factors such as cost, application needs, and detection time also influence design. Electrolytes like sodium, potassium, chloride and calcium are typically present in millimolar concentrations and are measured using ion-selective electrodes and potentiometric methods. In contrast, metabolites such as glucose and lactate occur at lower (sub-millimolar to micromolar) levels and are detected using enzymatic reactions with amperometric sensing. Detecting biomarkers at very low (sub-micromolar) concentrations remains difficult due to limited sensitivity and interference from other substances in sweat.^172^ Therefore, wearable glucose sensors must conform to the skin and withstand mechanical deformation. To achieve this, flexible and biocompatible substrates such as polyimide, polydimethylsiloxane, polyethene terephthalate, and textile fibres are used.^173,174^ MOFs can be integrated onto these substrates through *in situ* growth, drop-casting, printing, or embedding within polymer matrices.^175–178^ Integrating MOF materials onto a flexible substrate is a key strategy for developing high-performance flexible sensors. Common techniques such as drop casting, spin coating, and dip coating are favoured for their simplicity, low cost, and adaptability. These methods deposit pre-synthesised MOF crystals onto flexible materials like polymers or fabrics, creating stable and durable sensing platforms.^179–181^

Zha *et al.* developed a wearable non-enzymatic glucose sensor using 2D bimetallic Ni–Co MOF nanosheets. The MOF was drop-cast onto the working electrode and covered with a PVA/KOH gel layer, enabling the device to function both as a glucose sensor and a micro-supercapacitor for wearable applications. The sensor showed high sensitivity and effective glucose detection in sweat samples. Its enhanced performance is due to the synergistic interaction between Ni and Co ions, which creates unsaturated metal sites and defects, thereby increasing active reaction sites and improving glucose oxidation. A wearable version fabricated on a PET substrate using magnetron sputtering exhibited a glucose-sensing sensitivity of 0.31 µA µM^−1^.^182^ Similarly, Shu and coworkers developed a wearable Ni–Co MOF nanosheet-based glucose sensor for sweat analysis. A stretchable fibre electrode was fabricated using reduced graphene oxide and polyurethane through wet spinning, followed by coating with conductive silver and Ni–Co MOF nanosheets. The sensing system included an Ag/AgCl reference electrode and a platinum auxiliary electrode, both integrated onto a waterproof bandage with a sweat-absorbing layer (Fig. 8a). The sensor enabled continuous monitoring of sweat glucose in human subjects and showed reliable performance compared with a commercial glucose meter. The device demonstrated excellent mechanical stability, strong electrocatalytic activity, good selectivity, and acceptable long-term stability for wearable glucose sensing applications.^183^ In the same way, Yuan *et al.* developed a portable wearable sweat-based glucose sensor using Ni–Co MOFs integrated into a three-layer fabric device consisting of an accelerated diffusion layer, detection layer, and hydrophobic layer. Carbon ink was printed on nylon film to form the working and counter electrodes, while silver ink was used for the reference electrode. The Ni–Co MOF was coated onto the working electrode to improve electrocatalytic glucose sensing. The sensor exhibited a high surface area of 424.41 m^2^ g^−1^, pore volume of 0.00542 cm^3^ g^−1^, and glucose sensitivity of 2.935 µA mM^−1^, and a detection limit of 1.686 µM. The multilayer fabric efficiently transports sweat for real-time glucose monitoring^176^ as shown in Fig. 8b. Further, Xia and colleagues developed the Stamping Vacuum Filtration Dry Transfer (SVFDT) method to fabricate a flexible wearable electrochemical glucose sensor. Initially, a three-electrode pattern was engraved onto a PVC stamp and transferred onto filter paper using PDMS ink to create the electrode template. Multiwalled Carbon Nanotubes (MWCNTs) were then vacuum-filtered onto the patterned template and dry-transferred onto a PDMS substrate to form a flexible conductive MP electrode. To improve conductivity, an additional CNT conductive layer was stamped onto the electrode surface, producing a CMP electrode. Subsequently, NiCo MOF nanomaterials were drop-cast onto the working electrode to obtain the NCMP sensing platform. Finally, Ag/AgCl was coated as the reference electrode. A sweat-absorbing cloth was integrated to collect sweat directly from the skin. The fabricated sensor exhibited excellent analytical performance with a wide linear detection range of 20 µM^−1^ mM, high sensitivity of 71.62 µM^−1^ cm^−2^, and a low detection limit of 6.78 µM. The sensor was tested on volunteers before and after meals and successfully performed real-time glucose monitoring from sweat samples^184^ (Fig. 8c and d). Rebecca *et al.* reported a zeolitic imidazolate framework-8-based self-powered wearable glucose sensor using a hybrid of ZIF-8 MOF and reduced graphene oxide. The two materials were synthesised separately, mixed through ultrasonication, and then purified by washing, centrifugation, and drying to form the MOF/rGO electrocatalyst. This electrocatalyst was coated onto a graphite pencil electrode to evaluate its glucose-sensing performance. The sensor exhibited strong electrocatalytic activity, high sensitivity, and good stability for glucose detection, as shown in Fig. 8e. A two-electrode wearable patch sensor was subsequently fabricated and successfully used for sweat glucose monitoring, showing results that closely matched standard glucose meter readings.^185^ In another work, a ZIF-67-based wearable glucose sensor was developed using palladium nanoparticles (PdNPs) encapsulated within a Co-MOF. The Co-MOF was first synthesised, followed by the incorporation of PdNPs through an impregnation–reduction method to form Pd@Co MOF. This composite was mixed with conductive carbon ink and screen-printed onto a PET film to fabricate the working electrode. The counter electrode was also prepared using conductive carbon ink, while the reference electrode consisted of an Ag/AgCl electrode coated with a PVA/KCl polymer layer. Initially, the sensor showed no electrochemical redox activity, but pretreatment at −2.0 V for 20 S generated a temporary alkaline environment near the sensor surface, enabling glucose oxidation in sweat. The sensor was integrated into a wearable sweatband for real-time sweat glucose monitoring and demonstrated good agreement with conventional blood glucose measurements. Additionally, the sweatband was capable of transmitting real-time glucose data directly to a smartphone.^186^

**Fig. 8:**
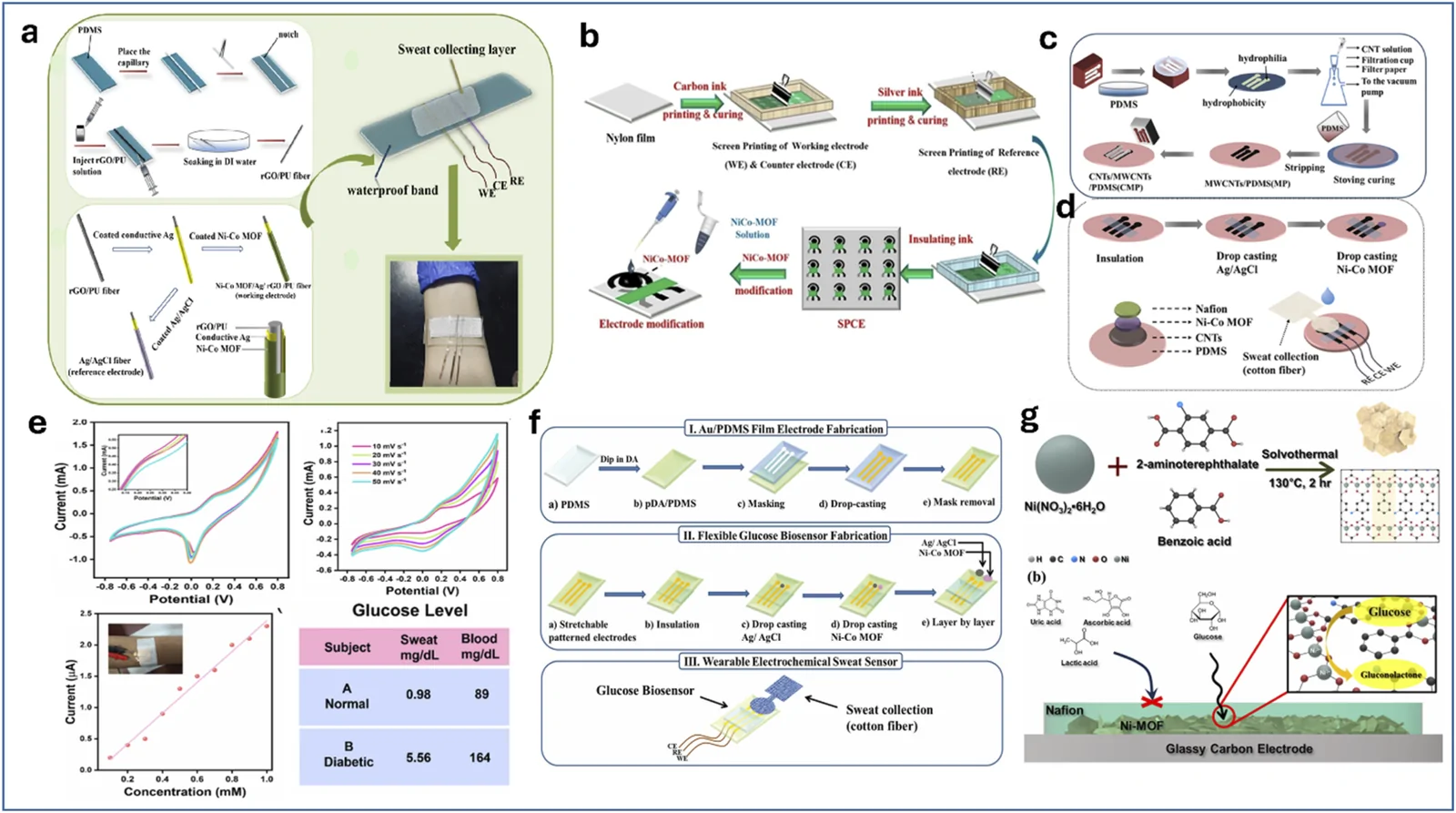
(a) Fabrication and integration of NiCo-MOF-coated rGO/PU fibre electrode into a wearable sweat-based glucose sensor worn on the arm. Reproduced from ref. 183 with permission from the American Chemical Society, copyright 2021. (b) Schematic representation of NiCo-MOF-modified SPCE fabrication on a nylon film. Reproduced from ref. 176 with permission from Wiley-VCH GmbH, Weinheim, copyright 2023. (c and d) Fabrication of NiCo-MOF-modified CNT/PDMS flexible electrode integrated with a cotton fibre sweat-collecting layer for a wearable glucose sensor. Reproduced from ref. 184 with permission from Elsevier B.V., copyright 2023. (e) CV response at varying glucose concentrations and scan rates, prototype current response to glucose concentration measured *via* a multimeter and correlation of sweat glucose levels with blood glucose values from a commercial glucometer for normal and diabetic subjects. Reproduced from ref. 185 with permission from the American Chemical Society, copyright 2025. (f) Fabrication of a wearable glucose sensor by depositing Au on a PDMS film and integrating with a cotton fibre sweat-collecting layer. Reproduced from ref. 187 with permission from Royal Society of Chemistry, copyright 2022. (g) Synthesis of Ni-MOF and its application as a Nafion-coated GCE for selective electrochemical glucose detection. Reproduced from ref. 188 with permission from Springer Nature, copyright 2026.

Uniform coating of MOFs using drop-casting, spin-coating, or dip-coating can be difficult because these methods are sensitive to environmental conditions and substrate orientation. To improve film uniformity and adhesion, *in situ* growth techniques such as solvothermal synthesis, hydrothermal synthesis, liquid-phase deposition, electrodeposition, oxidative recombination, and layer-by-layer assembly are widely used. These methods allow MOFs to grow directly on flexible substrates, producing stronger, more uniform, and highly adherent sensing films.^189^ Shu *et al.* designed a wearable electrochemical sweat glucose sensor by coating NiCo MOFs nanosheets onto a stretchable Au/PDMS electrode prepared through chemical gold deposition and solvothermal synthesis. The sensor showed excellent electrochemical performance, with a wide glucose detection range and high sensitivity. Its fabric-covered sensing area and secure attachment enabled stable, accurate, and continuous glucose monitoring throughout daily use^187^ (Fig. 8f). Similarly, Wei developed a flexible Co-MOF-based sensor using carbon cloth and paper substrates through a wax-dyeing technique. The resulting Co-MOF/CC/paper sensor showed improved structural strength and toughness compared to conventional glassy carbon electrodes. Its porous cellulose structure increased the active surface area and crystallinity, enhancing sensor performance, flexibility and mechanical durability for wearable sensing applications.^190^ Another study presents a defect-engineered Ni-based MOF for non-enzymatic glucose sensing, where benzoic acid is introduced as a secondary linker to create structural defects and enhance the Ni^3+^ active sites. For electrode preparation, the MOF suspension was drop-cast onto a glassy carbon electrode and coated with Nafion to improve selectivity, followed by electrochemical testing with a three-electrode system using 0.1 M NaOH electrolyte. Additionally, a flexible sensor was fabricated using screen-printed carbon and Ag/AgCl inks on a PET substrate, demonstrating scalability and compatibility with wearable applications (Fig. 8g). Among the samples, Ni-MOF-10 exhibits the best performance with high sensitivity and selectivity over a wide linear concentration range of 0.5–2 mM, with a linear correlation coefficient of 0.9997. These findings demonstrated that altering the chemical environment in Ni-MOF can significantly improve glucose sensitivity.^188^

Further, Table 2 shows the electrochemical performance of a MOF-based wearable and portable glucose sensor.

**Table 2:** Electrochemical performance of MOF-based wearable and portable Glucose sensor

Sensors	Linear range	pH	LOD (µM)	Potential (V)	Sensitivity	Ref.
PVA/KOH/NiCo-MOF/GCE	5–205 µM	Basic	0.11	0.50	1422.2	182
	205–2655 µM				522.9	
	2655–5655 µM				285.8 µA mM^−1^ cm^−2^	
PVA/KOH/NiCo-MOF/PET	10–200 µM	Basic	10	0.50	0.31 µA µM^−1^	182
NiCo-MOF/Ag/rGO/PU	10–0.66 mM	7.0	3.28	0.50	425.9 µA mM^−1^ cm^−2^	183
NF/NiCo/CNTs/MWCNTs/PDMS	20–1.1 µM	Basic	6.78	0.50	71.62 µA mM^−1^ cm^−2^	184
ZIF-8/rGO/PGE	0.005–5 mM	7.4	0.3	0.35	5047.1 µA mM^−1^ cm^−2^	185
NiCo/Au/PDMS film	20–790 µM	7.0	4.25	0.55	205.1 µA mM^−1^ cm^−2^	187
NiCo-MOF/NF	0.04–5.34 mM	Basic	1.686 mM	0.60	2.935 µA mM^−1^	176
NiMn-MOF	0–0205 mM	Basic	0.28	0.50	1576	191
	0.255–2.655 µM				1285	
	3.655–5.655 µM				755.7 µA mM^−1^ cm^−2^	
CeO_2_NPs/Ni-MOF	40–1200 µM	Basic	30	0.55	2488 µA mM^−1^ cm^−2^	192

### 6.2 Sweat collection and microfluidics

Efficient sweat collection and transport are critical for reliable sensing. Microfluidic channels, hydrogels, and capillary-driven structures are commonly integrated with MOF-based electrodes to ensure controlled delivery of sweat to the sensing interface. Sweat samples can be collected in two main ways: one is passive, and the other is active. The passive approach relies on physical activity, such as running or cycling, to naturally produce sweat. The active approach induces sweat without exercise, typically using electrical stimulation. A common active method is iontophoresis, which enables sweat collection while the person remains at rest. Iontophoresis is a widely used technique for inducing sweat, where an applied voltage generates a current beneath the skin.^169^ This current drives agonist molecules such as pilocarpine into sweat glands, stimulating sweat secretion. This method has been effectively used for monitoring biomarkers like chloride, ethanol and glucose.^193^ A wearable sweat alcohol biosensing device based on iontophoresis introduces further improvements to maintain the integrity of sweat samples. The device incorporates a membrane to isolate sweat irritants from skin, preventing dilution of analytes. Additionally, sudomotor axon reflex sweating minimises the mixing of fresh and previously secreted sweat and reduces contamination. The use of carbachol as an agonist ensures a longer and more stable sweat production rate. Furthermore, a hex wick material efficiently transports sweat to the sensor, preventing skin contamination and reducing response time. However, conventional iontophoresis techniques can lead to electrode corrosion and skin irritation, causing discomfort.^194^ To address these issues, a programmable current source is implemented to control and periodically induce sweating, while maintaining an upper current limit as a safety mechanism. This approach helps to prevent overheating and skin damage, ensuring safer and more reliable operation.^171,195,196^

Yang, *et al.* introduce a wearable wireless patch that integrates microfluidic technology with enzyme-free electrochemical sensing for real-time glucose monitoring in sweat. The system utilises a microfluidic channel to autonomously collect and transport sweat, in which *in situ* chemical processing occurs *via* dissolution of solid NaOH to create the alkaline environment required for nanozyme activity. An Au-NPS/Ni-BDC MOF-based sensing layer ensures high sensitivity and stability without depending on biological enzymes. To enhance operational safety, a Tesla valve is incorporated to maintain unidirectional flow and prevent backflow of alkaline solution onto the skin. Furthermore, an integrated pH and temperature sensor enables continuous calibration, compensating for environmental variations and improving measurement accuracy. The device is coupled with a wireless communication module, allowing real-time data transmission to a smartphone for continuous health monitoring^197^ (Fig. 9a) Likewise, Bae *et al.* developed a fully stretchable passive capillary microfluidics system integrated with a wearable glucose biosensor for continuous glucose monitoring. This system employs hydrophilic and stretchable textile materials, such as cotton fabric, to facilitate sweat transport through capillary action without requiring external pumps or additional power sources. The capillary fabric is typically embedded in elastomeric substrates such as polydimethylsiloxane (PDMS) reinforced with polyurethane nanofibers, which enhances mechanical durability, flexibility and conformal skin attachment^198^ (Fig. 9b). Further, Xu *et al.* introduce a wedge-shaped microfluidic channel and double-layer capillary micropump to improve passive sweat transport and collection efficiency. They also developed a highly sensitive non-enzymatic Cu-MOF/PANI electrochemical sensor for stable glucose detection, along with Na^+^ and K^+^ sensing^199^ (Fig. 9c). Similarly, Yin *et al.* developed a wearable microfluidic device that enhanced sweat transport by employing optimised PDMS microchannels simulated using COMSOL software. Proper channel sizing improved sweat flow efficiency, reduced pressure resistance, and enabled continuous directional transport of fresh sweat. This approach minimised evaporation and contamination, thereby improving the reliability and accuracy of real-time electrochemical detection of multiple sweat biomarkers^200^ (Fig. 9d).

**Fig. 9:**
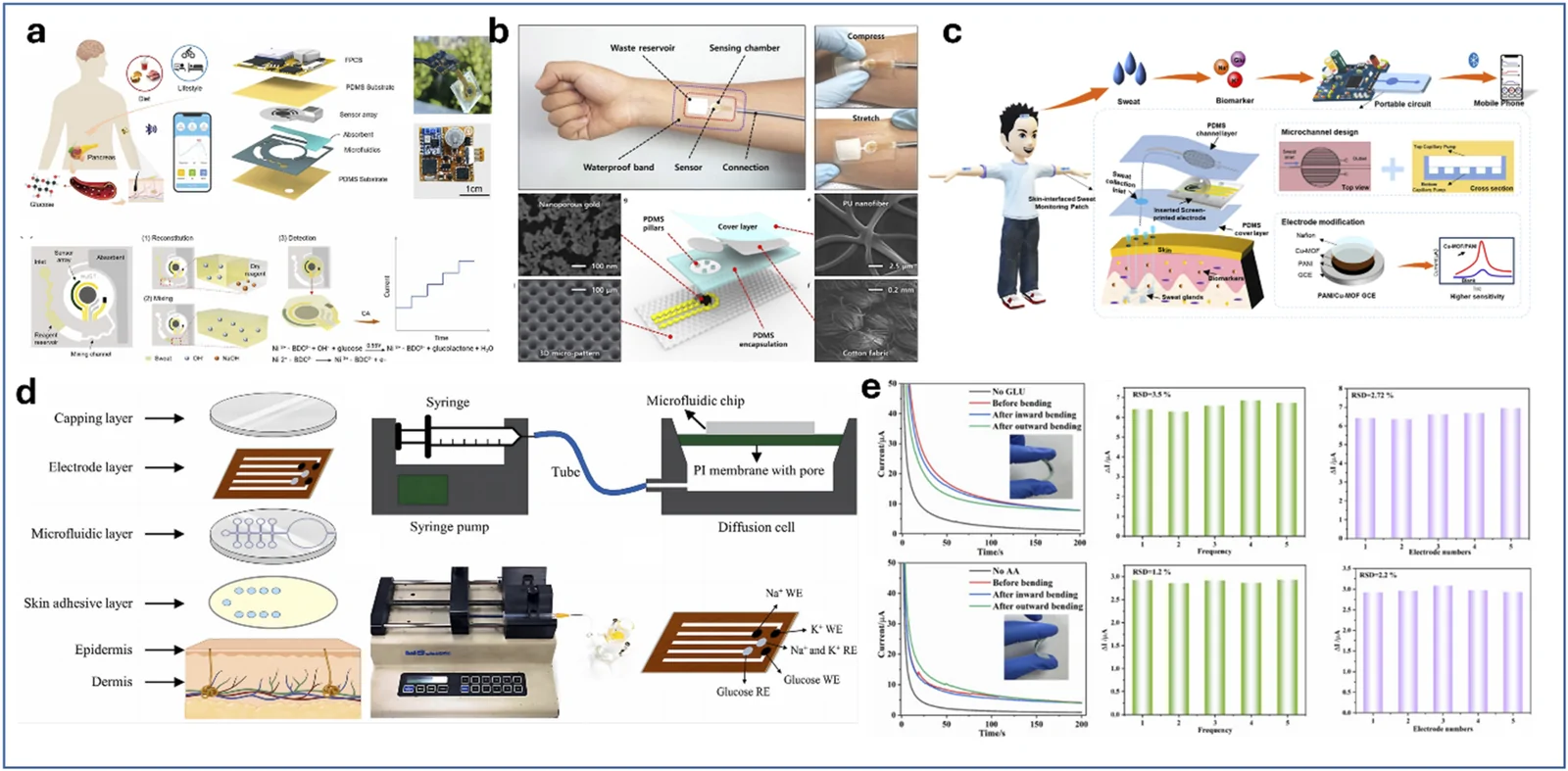
(a) A wearable enzyme-free microfluidic patch integrating a Ni-BDC sensor array on a flexible PDMS substrate for wireless real-time sweat glucose. Reproduced from ref. 197 with permission from the American Chemical Society, copyright 2025. (b) A stretchable wearable biosensor patch integrating nanoporous gold electrodes, a 3D micropatterned PDMS substrate, PU nanofiber and a cotton fibre layer for conformal skin-mounted sweat sensing. Reproduced from ref. 198 with permission from the American Chemical Society, copyright 2019. (c) Wearable PDMS-based sweat patch with PANI/Cu-MOF modified electrodes and bluetooth-enabled portable circuit for real-time monitoring of glucose, K^+^ and Na^+^. Reproduced from ref. 199 with permission from Elsevier B.V., copyright 2025. (d) Schematic representation of a wearable sweat microfluidic sensor system comprising a multilayered skin adhesive, an artificial sweat setup with a syringe pump and PI membrane diffusion cell, and an electrochemical sensor array for simultaneous detection. Reproduced from ref. 200 with permission from Elsevier B.V., copyright 2024. (e) Anti-bending, repeatability and reproducibility performance of the Cu@PHEMa/SPE electrode for electrochemical detection of glucose and ascorbic acid. Reproduced from ref. 201 with permission from Elsevier B.V., copyright 2025.

However, hydrogels are soft, water-rich materials used in flexible wearable sensors because of their biocompatibility, stretchability, and conductivity.^202^ To improve their performance, researchers combine hydrogels with conductive materials to create conductive hydrogels. Incorporating MOF into hydrogels strengthens the hydrogel network by acting as a crosslinking and reinforcement agent. MOFs also enhance electron transfer and electrical conductivity, overcoming the naturally low conductivity of traditional MOFs.^197^ The following passage describes two major methods for synthesising MOF-hydrogel composites.

(1) Direct incorporation of pre-synthesised MOFs:

MOF crystals are first synthesised separately and then mixed into hydrogel precursor solutions before gelation. During hydrogel formation, the MOFs become uniformly embedded within the hydrogel network, improving particle dispersion and interactions.^203,204^

(2) *In situ* growth of MOFs inside hydrogels:

MOF precursors are first introduced into an already formed hydrogel matrix. MOFs then grown within the hydrogel pores through nanoconfinement effects or external stimuli. This method promotes stronger synergistic interactions between MOFs and hydrogels through chemical or physical interconnection and deposition processes. Overall, both strategies enable control over the structure, composition, and properties of MOF-hydrogel composites, allowing optimisation for specific applications.^205–208^

Therefore, Yue *et al.* developed a d-π conjugation strategy to modify 2D cobalt MOFs, thereby significantly enhancing the electrical conductivity of cellulose-based hydrogels and enabling highly sensitive self-powered strain sensing. The incorporation of MOFs improved electron-transfer kinetics, overcoming the intrinsic low conductivity and poor carrier mobility of conventional MOFs and further enhancing hydrogel conductivity. In addition, the integration of MOFs with hydrogel exploited the multifunctional nature of MOFs to tailor the structural and functional properties of the hydrogel matrix.^209^ Further, Wang Fang *et al.* developed a flexible Cu@PHEMA hydrogel-based electrochemical sensor based on a copper-doped poly(2-hydroxymethyl methacrylate) hydrogel integrated with a screen-printed electrode for glucose and ascorbic acid detection. The hydrogel was synthesised through a simple free-radical polymerisation process using Cu^2+^/tannic acid redox polymerisation method. Mechanically, the hydrogel exhibited excellent tensile strength of 184 kPa, elongation at break of 346%, and retained 85% of its tensile strength after 50 loading and unloading cycles, indicating strong fatigue resistance. It also showed self-adhesive strength of 96 kPa on pig skin and 54 kPa on PMMA surfaces. Functionally, the sensor demonstrated high sensitivity and low detection limits. For glucose, the detection range was 0–2 mM with sensitivities of 122.24 and 24 µA mM^−1^ cm^−2^ and a detection limit of 4.5 µM. For ascorbic acid, the range was 0–0220 µM, with sensitivities of 799.85 and 201.94 µA mM^−1^ cm^−2^ and a detection limit of 0.96 µM. The sensor also exhibited excellent flexibility, selectivity, stability and accurate real sweat analysis^201^ (Fig. 9e).

### 6.3 Mechanical stability and wearability

The mechanical integrity of MOF-based sensors under bending, stretching, and repeated use is a key consideration. Composite designs and encapsulation strategies are often employed to enhance durability without compromising sensing performance.

Zha *et al.* developed a wearable non-enzymatic glucose sensor using 2D bimetallic Ni–Co MOF nanosheets. The nanosheet network holds up to mechanical deformation while maintaining continuous conductive pathways and electrochemical activity. Its flower-like architecture distributes stress uniformly, preventing structural damage during movement. The sensor could closely attach to the skin and operate effectively under different bending conditions. Even at a bending angle of 180°, the oxidation peak current decreased by only 6.94%, confirming strong mechanical durability. These properties make the electrode highly suitable for wearable applications^182^ (Fig. 10a) Shu *et al.* developed a Ni–Co MOF/Ag/rGO/PU fibre electrode that exhibited excellent stretching and bending performance, making it highly suitable for wearable glucose sensing applications. Under tensile strain from 0–100%, the oxidation peak current decreased by only about 19.9%, demonstrating strong electrochemical stability during stretching. Even after 10 000 stretching cycles at 20% strain, the redox peak current showed almost no significant change, confirming excellent mechanical durability and repeatability. The electrode also maintained stable performance under bending angles from 30° to 90°, with only resistance and current variations. These results indicate that the flexible fibre electrode can sustain continuous electrochemical activity under severe mechanical deformation.^183^ Yuan *et al.* developed the NiCo-MOF wearable sensor, which exhibited excellent stretching, bending, and mechanical stability due to its flexible multilayer fabric structure and interconnected porous nanosheet architecture. The flexible fabric substrate allowed the sensor to conform closely to the human body while maintaining continuous electrochemical performance during movement and deformation. Strong hydrogen bonding between the MOF coating and the fabric fibre improved adhesion and durability during repeated mechanical deformation and washing. These properties ensured stable sensing performance, reduced signal drift and reliable long-term wearable glucose monitoring^176^ (Fig. 10b). In another work, Feng *et al.* developed a flexible non-enzymatic electrochemical glucose sensor based on NiCo-MOF nanosheets integrated onto an OPU-coated polypropylene spun-bonded nonwoven fabric (PPFS) substrate for continuous sweat glucose monitoring. The sensor demonstrates excellent mechanical flexibility, which is essential for wearable applications. Tensile testing showed that the elongation at break increased from 80.22% for PPSE to 152.44% after sensor fabrication, indicating high elasticity and durability. The bending stability revealed only minor signal variation under bending angles up to 300°, confirming stable electrochemical performance during deformation. Temperature stability studies between 24 and 40 °C showed nearly overlapping current responses, indicating reliable glucose sensing under physiological temperature variations. These properties make the sensor highly promising for wearable diabetes monitoring applications^210^ (Fig. 10c) Correspondingly, Xia and colleagues reported that the Ni–Co MOF/CNTs/MWCNTs/PDMS (NCMP) film electrode showed excellent mechanical stability under both stretching and bending conditions. Cyclic voltammetry (CV) tests demonstrated that the electrode maintained stable electrochemical performance even when stretched from 0% to 8% strain. The oxidation peak current decreased by only about 21.2% at 8% tensile strength, indicating good stretchability and conductivity retention. For bending stability, the electrode was repeatedly bent up to 100 times, and almost no significant change in oxidation peak current was observed, confirming strong bending durability. Additionally, the wearable sensor was tested in both bent and non-bent states at different glucose concentrations (0.6, 0.7 and 0.8 mM). The maximum difference in current response between the two conditions was less than 3%, further proving the high mechanical stability and reliability of the sensor during deformation.^184^ Asaduzzaman *et al.* developed a flexible skin-integrated patch for simultaneous sweat biomarker analysis and ECG monitoring. The device combined MOF-derived hydroxy-functionalized hybrid nanoporous carbon with laser-scribed graphene. The nanoporous carbon provided a high surface area and strong electrochemical performance, enabling sensitive detection of sweat biomarkers such as glucose and uric acid. Laser-scribed graphene improved flexibility and ensured close skin contact for reliable signal collection. The wearable patch showed stable, durable, and effective real-time performance for both sweat sensing and ECG monitoring.^211^

**Fig. 10:**
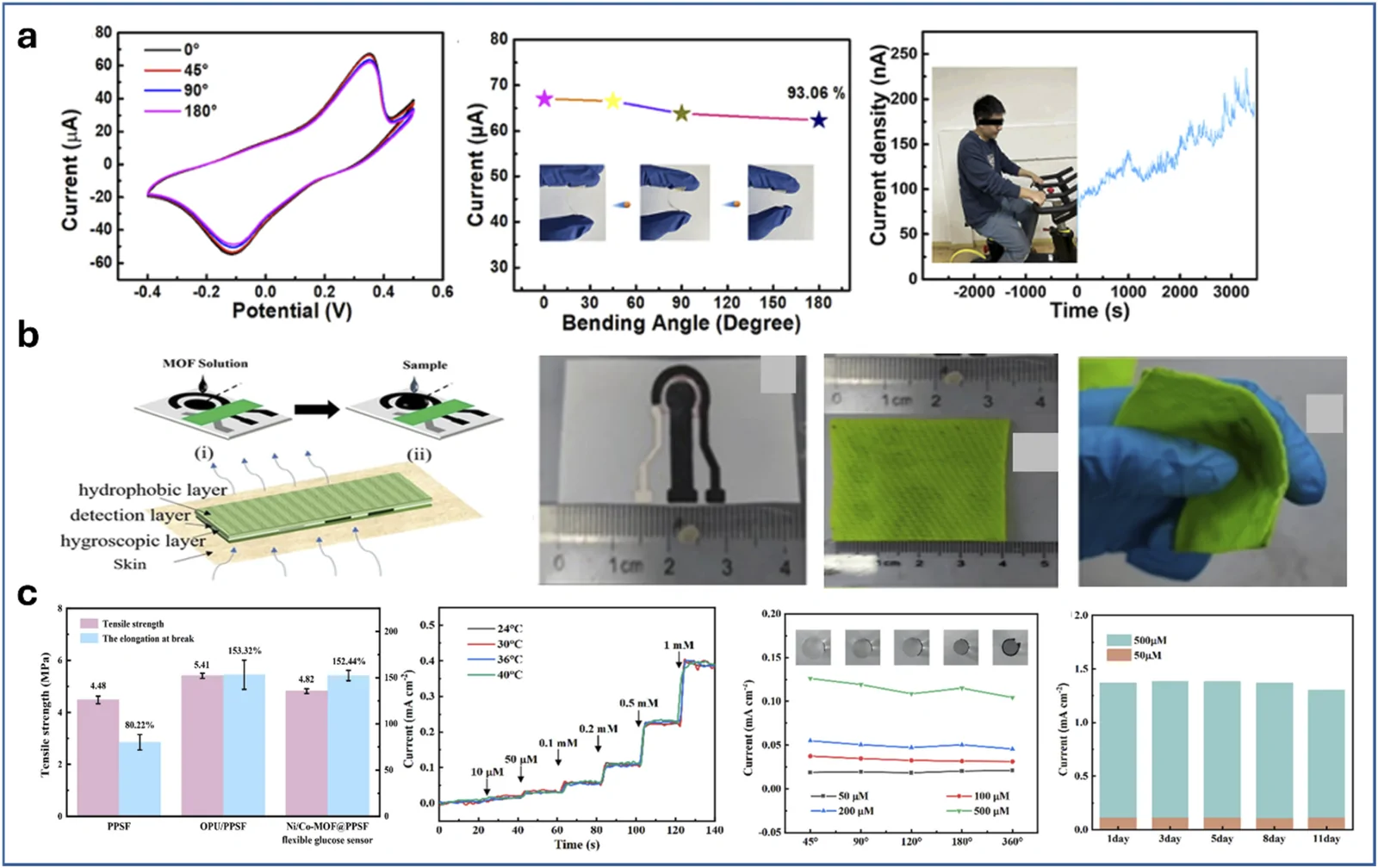
(a) CV stability of the glucose sensor at various bending angles with 93.06% current retention and real-time sweat glucose monitoring during exercise. Reproduced from ref. 182 with permission from Royal Society of Chemistry, copyright 2023. (b) A multilayer biosensor comprising hydrophobic, detection, and hygroscopic layers, fabricated *via* MOF drop-casting, is demonstrated using schematic diagrams and photographs of flexible bending. Reproduced from ref. 176 with permission from Wiley-VCH GmbH, Weinheim, copyright 2023. (c) The mechanical properties of the NiCo-MOF@PPSF flexible glucose sensor, including tensile strength and elongation, demonstrate stable electrochemical performance across varying temperatures, bending angles, and storage conditions. Reproduced from ref. 210 with permission from the American Chemical Society, copyright 2026.

### 6.4 Self-powered MOF-based wearable systems

Self-powered wearable biosensors have emerged as a promising strategy for continuous an autonomous health monitoring by reducing or eliminating the dependence on conventional external batteries. In this context, MOFs are attractive materials for developing self-powered sensing platforms because of their specific surface area, tunable pore structure, diverse metal centres, and readily tailorable chemical functionalities. These characteristics allow MOFs to be integrated with energy harvesting and energy storage materials while simultaneously providing electroactive interfaces for biosensing. Among the different approaches, triboelectric nanogenerators (TENGs), biofuel cells, and hybrid energy storage have attracted considerable attention for powering wearable sensors.

The potential of MOFs for mechanical energy harvesting was initially demonstrated through MOF-based triboelectric nanogenerators. Khandelwal *et al.* reported a ZIF-8-based triboelectric nanogenerator for self-powered sensing applications, demonstrating that the porous MOF structure can function as an effective triboelectric material. The ZIF-8/Kapton-based TENG generated an output voltage of approximately 164 V and a current of 7 µA, demonstrating the feasibility of using MOFs for converting mechanical energy into electrical energy. This study established an important foundation for the development of MOF-enabled self-powered sensing systems, in which energy generated from body motion or environmental mechanical stimuli can potentially replace conventional power sources.^212^ Subsequently, studies have extended MOF-based triboelectric systems toward wearable healthcare applications. Rana *et al.* developed a MOF-525/α-MoO_3_ hybrid systems exhibited a power density of 18.38 W m^−2^ and enabled the detection of various biosignals and biomechanical activities, including body motion, respiration, pulse, and muscle-related signals. The incorporation of MOF-525 into the hybrid triboelectric architecture provided a high-surface-area porous interface, while the incorporation of α-MoO_3_ contributed to charge generation and transport. Such MOF-based hybrid TENGs demonstrate the possibility of harvesting mechanical energy generated during routine human activities and directly utilising it for wearable healthcare monitoring.^213^ Beyond mechanical energy harvesting, the integration of MOFs with electrochemical glucose sensing electrodes provides another important route toward self-powered wearable glucose monitoring. In particular, hybridisation of MOFs with conductive carbon materials such as reduced graphene oxide (rGO) can address the relatively poor electrical conductivity of many pristine MOFs while retaining their high surface area and abundant active sites. A recent study based on Cu-BDC/rGO heterostructures demonstrated a self-powered, non-enzymatic glucose sensing platform with high sensitivity and selectivity. The Cu-BDC MOF provided electroactive metal centres and a porous structure favourable for glucose oxidation, whereas rGO facilitated electron transport and improved electrical conductivity of the hybrid electrode. The resulting system was further integrated with energy-storage and wireless communication components, enabling real-time glucose monitoring without relying on an external power supply. This work illustrates the potential of combining MOF-based electrocatalysis with energy-management components to construct autonomous glucose-monitoring systems.^214^

These hybrid architectures are highly promising for wearable sweat-glucose monitoring because body movements can provide mechanical energy through TENGs. Integrating a MOF-based non-enzymatic glucose electrode with a TENG and microsupercapacitor could enable energy harvesting, storage, sensing, and wireless communication in a compact, battery-free device.

However, challenges remain, including intermittent power generation, limited energy-storage efficiency, electrode instability in sweat, biofouling, and interference from electroactive metabolites. Further improvements in MOF stability, catalytic selectivity, flexible integration, energy management, and sweat-compatible design are required to achieve reliable autonomous glucose-monitoring platforms.

## 7. Electrochemical performance metrics

Electrochemical sensing has emerged as a leading approach for wearable sweat analysis because of its high sensitivity, selectivity, rapid response, low power requirements, and compatibility with flexible and wearable platforms. Several electrochemical techniques have been explored for the detection of biomarkers, electrolytes, metabolites, hormones, drugs and toxic substances in sweat, including potentiometry, chronoamperometry, cyclic voltammetry (CV), differential pulse voltammetry (DPV), square wave anodic stripping voltammetry (SWASV), and electrochemical impedance spectroscopy (EIS).

Potentiometric sensing is one of the most widely used methods for monitoring ionic species in sweat. This technique measures the change in electrode potential that occurs in response to variation in analyte concentration and has been successfully applied for the detection of major sweat electrolytes such as sodium, potassium, and calcium.^215–217^ Due to its simplicity and low power consumption, potentiometry is particularly attractive for continuous wearable monitoring.

Chronoamperometry is commonly employed in enzyme-based biosensors. In this method, a constant potential is applied, and the resulting current generated from the analyte-specific redox reaction is measured. Enzymatic sensors for glucose monitoring represent a prominent example, where glucose oxidase catalyses glucose oxidation, producing an electrical signal proportional to glucose concentration.^193,218^ Advanced electrochemical techniques, including CV, DPV, SWASV, and EIS, have expanded the scope of wearable sensing to cover heavy metals, drugs, hormones, proteins and other biomarkers.^219–222^ Voltammetric methods operate by sweeping the electrode potential across a defined range and measuring the resulting redox currents. Among these techniques, CV is widely used for sensor characterisation, providing insights into electron transfer kinetics and redox mechanisms, while also enabling the detection of metabolites such as glucose, uric acid, and ascorbic acid.^220^ DPV offers enhanced sensitivity and is frequently utilised for detecting organic and inorganic analytes, particularly proteins, through surface binding interactions.^223,224^ SWASV is highly effective for trace-level metal analyses because it incorporates a preconcentration step that significantly enhances detection sensitivity. In addition to their advantages, voltammetric techniques face challenges such as overlapping redox potentials, interference from coexisting species, and the formation of intermetallic compounds that can compromise analytical performance. EIS, on the other hand, detects bioaffinity interactions by monitoring changes in interfacial impedance and analysing the resulting Nyquist plots. Although EIS provides a label-free and highly sensitive detection platform, it requires longer measurement times and advanced data processing to minimise uncertainty.^225^ In general, the diverse range of electrochemical sensing modalities enables comprehensive sweat analysis, with each technique offering distinct advantages depending on the target analyte and based on the application requirements. Their integration into wearable devices continues to focus on advancements in non-invasive health monitoring and personalised diagnostics.^216,226,227^Table 3 comprises the selection criteria for electrochemical sensing modalities based on target characteristics. Following that (Table 4), summarises the various MOFs used for electrochemical sensing techniques depending on the target analyte molecules.

**Table 3:** Selection criteria for electrochemical sensing modalities based on target characteristics

Electrochemical technique	Working principle	Advantages	Limitations	Ref.
Potentiometry	Measures the potential difference between sensing and reference electrodes, which correlates with ion activity or concentration	Simple instrumentation and signal processing, suitable for ionic species with stable charge states, effective for analytes in the mM concentration range	Requires ion-selective membranes for specificity, susceptible to interference from competing ions, and limited to charged analytes	215
Voltammetry	Measures current response during a controlled potential sweep to obtain analyte-specific redox information	Enables simultaneous detection of multiple analytes, offers diverse measurement modes for signal optimisation, and achieves high sensitivity through pre-concentration strategies	Background redox processes can interfere with signals, require more complex data analysis and peak interpretation	193
Chronoamperometry	Applies a constant potential and monitors the resulting current generated by redox reactions, proportional to analyte concentration	Rapid and straightforward quantification, low power requirements when redox mediators are used, compatible with portable sensing platforms	Signal decay may reduce the accuracy of the trace concentrations, and selectivity often depends on biorecognition elements such as enzymes	216, 219, 224 and 225
Electrochemical impedance spectroscopy	Determines changes in impedance at the electrode interface caused by target binding events	Supports label-free detection, highly suitable for affinity-based biosensing, sensitive to surface interactions and binding kinetics	Longer measurement limits, computationally intensive data analysis, often require signal amplification to improve sensitivity	222 and 225

**Table 4:** This summary highlights recent progress in using metal–organic frameworks for electrochemical sensing modalities depends on target characteristics

MOF-based sensors	Technique used	Analytes	Linear range	LOD	Sensitivity	Ref.
UiO-66-NHC(S)NHMe/3D-KSC	SWASV	Cd^2+^, Pb^2+^, Cu^2+^, and Hg^2+^	0.0372–8.0, 0.0333–8.0, 0.0375–8.0 and 0.0282–8.0 µM	0.0125, 0.0124, 0.0111, and 0.0094 µM	879.1, 887.6, 992.5, and 1160.7 µA µM^−1^ cm^−2^	228
Co@NC/MWCNT	SWSV	Cd^2+^, Pb^2+^	0.12–2.5 µM	4.5 nM, 4.9 nM		229
Au@Cu-MOF	DPV	Glutathione	0.01–40 nM	2.535 pM		230
NH_2_-MIL-101(Fe)/CNF@AuNPs	EIS	Tetracycline	0.1–10^5^ nM	0.01 nM		231
MOF (GCE/CAU-1)	Amperometry	Hydroquinone	0.5–1500 µM	0.067 µM	1471.4 µA mM^−1^ cm^−2^	232
Ni_2_P/C	DPV	Uric acid	0 to 6.0 µM	7.0 × 10^−8^ mol L^−1^	0.552 µA µM^−1^	233
W-ZIF-67	DPV	Uric acid	20–1000 µM	3.04 µM	0.0377 µA µM^−1^ cm^−2^	234
POMOF	DPV	Xanthine	0.5–240 µM	0.26 µM	2.05 µA µM^−1^ cm^−2^	235
3D MOF-derived Co_3_O_4_@C/GNP	DPV	Metol, hydroquinone and catechol	0.01–80 µM, 0.04–30 µM and 0.5–30 µM	5.1, 14.7 and 169 nM		236
BC/Cr_2_O_3_/Ag/MIP/GCE	DPV	Nitrofurazone	0.005–10 µM	0.003 µM		237
GN/ZIF-8	DPV	8-Hydroxy-2′-deoxyguanosine, acetaminophen and benomyl		1.58 nM, 7.50 nM and 2.10 nM	270.00 µA µM^−1^ cm^−2^, 757.14 µA µM^−1^ cm^−2^ and 272.86 µA µM^−1^ cm^−2^	238
CNT/Ni-MOF-74 derived 0.2CNT/NiSe_*x*_	DPV	Acetaminophen and dopamine	1–600 µM and 1–500 µM	0.55 and 0.72 µM		239
Cu-MOF/CuO/NiO NCs	DPV	Catechol	0.01–22 µM	0.0078 µM		240
Ni–Co MOF/Ag/rGO/PU	Amperometry	Sweat glucose	10–0.66 mM	3.28 µM	425.9 µA mM^−1^ cm^−2^	183

Recent studies have demonstrated that metal–organic frameworks (MOFs) and their derivatives are promising materials for enzyme-free electrochemical glucose sensing. Under alkaline conditions, MOFs exhibit significant electrocatalytic activity toward glucose oxidation due to the redox-active metal centres (Mn^+^/Mn^+^ clusters) present within their frameworks. In addition, their abundant functional sites enhance selective glucose recognition, while their large surface area and high porosity improve detection sensitivity through efficient host–guest interactions. Despite these advantages, several challenges limit the practical implementation of MOF-based nonenzymatic glucose sensors.

[1] Dependence on alkaline media: most MOF-based glucose sensors require alkaline operating conditions, restricting their applicability in physiological environments and hindering the development of commercial and wearable sensing devices.^241^

[2] Limited selectivity and interference resistance: these sensors often operate at relatively high detection potentials, making them susceptible to interference from electroactive species such as dopamine, ascorbic acid, and uric acid. Furthermore, the influence of common physiological ions (Na^+^, K^+^, Mg^+^, and Ca^2+^) on sensor performance has not been thoroughly explored.^242^

[3] Stability, conductivity and scalability issues: although MOFs are widely employed for glucose electrocatalysis, their structural fragility can lead to performance deterioration during repeated use. Additional limitations include poor chemical stability and low intrinsic electrical conductivity. Moreover, complex synthesis procedures, reproducibility challenges, and high production costs hinder large-scale commercialization.^243,244^

However, future research focuses on improving operation under physiological conditions, enhancing selectivity and anti-interference performance, increasing structural stability and conductivity, and developing scalable, cost-effective synthesis strategies to facilitate real-world applications.

## 8. Biocompatibility and safety considerations

Biocompatibility is a critical requirement for skin-mounted MOF-based sensors because prolonged skin contact may cause irritation, inflammation, or toxicity due to possible leaching of metal ions from the framework. To address these issues, MOF materials are commonly encapsulated within biocompatible polymers or hydrogels such as polyvinyl alcohol (PVA), polyethene glycol (PEG), PDMS, chitosan and alginate. These materials create a protective interface between the sensor and skin while maintaining flexibility, conductivity, and mechanical stability. Hydrogels are particularly advantageous because of their soft, tissue-like nature, high water content, and ability to reduce mechanical mismatch with human skin. Studies have shown that MOF hydrogel composites improve sensor stability, comfort, and long-term wearability in epidermal sensing applications.

Systematic biocompatibility evaluation is essential before clinical translation. Cytotoxicity assessments, including MTT and live/dead assays, are used to evaluate the effects of released materials on cell viability, while skin-irritation and sensitisation studies assess inflammatory response during prolonged use. According to ISO 10993 medical device standards, such evaluations are mandatory for wearable biomedical devices. Furthermore, toxicity depends on the MOF composition, with Zn–Fe-based MOFs generally considered safer than Co or Cu based frameworks.

## 9. Challenges and opportunities

Despite the major progress made in MOF-based wearable glucose sensors, there are still several obstacles that need to be overcome before they can be widely used and accepted in the medical field. These challenges are not only related to the limitations of the use of materials but also to the difficulties of measuring glucose levels through sweat, which is a complex physiological process.

Although MOF-based frameworks have demonstrated excellent electrocatalytic activity toward non-enzymatic glucose oxidation, their long-term performance in wearable sweat environments remains an important challenge. Ni–Co MOF nanosheet-based electrodes integrated with flexible Au/PDMS substrates have been reported for continuous sweat glucose monitoring, showing high sensitivity, mechanical flexibility, and short-term stability during on-body measurements.^187^ Similarly, bimetallic Ni–Co MOF-coated CNT/PDMS electrodes have enabled real-time sweat glucose detection with a low detection limit of 6.78 µM and good stability under stretching and bending conditions.^184^ However, these studies primarily emphasise electrocatalytic activity, mechanical flexibility, and analytical performance, while the effects of prolonged exposure to complex sweat matrices and skin-derived contaminants require further attention. Sweat biofouling can arise from the accumulation of lipids, proteins, salts and other biological components on the electrode surface, potentially blocking active sites and causing signal drift. Therefore, antifouling interfaces based on hydrophilic, PEG or zwitterionic polymers could be integrated with MOF electrodes to minimise nonspecific adsorption while preserving access of glucose and hydroxide ions to the electroactive MOF sites. In addition, self-healing or self-cleaning coatings could compensate for mechanical damage and progressive surface contamination during prolonged use. Microfluidic architecture provides another promising strategy by continuously transporting freshly secreted sweat across the MOF sensing interface, reducing sample stagnation and contaminant accumulation. Recent MOF-based sweat platforms have demonstrated the feasibility of integrating MOF sensing materials with flexible microfluidic or sweat collection architectures, indicating a pathway toward more stable and field-deployable glucose sensors. Further MOF-based wearable glucose sensors should therefore combine high-performance electrocatalytic MOFs with antifouling coatings, continuous sweat management, environmental compensation, and regeneration strategies to achieve reliable long-term operation under realistic physiological conditions. A major research gap is the limited integration of antifouling engineering with MOF-based glucose electrodes. Most reported MOF-based wearable glucose sensors focus on improving surface area, redox activity, conductivity, flexibility, and glucose sensitivity, whereas systematic evaluation of biofouling during prolonged skin contact is comparatively limited. Further design could address this limitation by constructing hierarchical MOF/polymer interfaces, in which the MOF provides abundant catalytic sites while an ultrathin zwitterionic or PEG-based layer suppresses nonspecific adsorption without substantially hindering glucose diffusion. The incorporation of microfluidic sweat channels could further provide controlled and continuous transport of fresh sweat to the MOF electrode, while self-healing or electrochemically regenerated interfaces could periodically restore the active surface. Such multifunctional architectures highlight the short-term analytical sensitivity towards operational stability, biofouling resistance, and continuous monitoring, which are essential requirements for translating MOF-based wearable glucose sensors from laboratory demonstrations to practical long-term health-monitoring devices.

## 10. Future perspectives

Healthcare has emerged as one of the most important globally needed conditions due to the increasing burden of infectious and chronic diseases. Conventional diagnostic and therapeutic approaches for large populations are often expensive, time-consuming, and predominantly invasive, posing risks such as tissue injury and leading to secondary infections. Additionally, many existing diagnostic techniques depend on bulky and costly instrumentation, require trained personnel, and are usually limited to major medical centres, which hinders quick on-site testing. However, recent progress in flexible and portable electronics has spurred the creation of wearable and point-of-care sensors that provide non-invasive, affordable, and continuous health tracking in real-time applications. Among these technologies, electrochemical wearable and point-of-care sensors have attracted significant attention owing to their high sensitivity, selectivity, rapid response, portability, and adaptability for customised healthcare applications.

Among the diverse electrode materials investigated for electrochemical sensing, metal organic frameworks have become standout options. Their appeal lies in their highly porous, crystalline structures, significant surface areas, and ability to customise their chemical properties. Furthermore, MOFs support large-scale, eco-friendly manufacturing, making them practical for real-world sensor production. While their unique characteristic have proven effective in energy and general sensing, their integration into wearable healthcare platforms remains an area with significant growth potential, as relatively few studies currently exist. Therefore, metal organic frameworks significantly enhance sensor performance because their large surface areas and adjustable pores allow for superior analyte adsorption and the precise housing of biomolecules. By engineering their internal chemistry, researchers can further boost catalytic activity and molecular recognition. Additionally, the ability to synthesise MOFs sustainably makes them a viable option for affordable healthcare technologies. However, moving from lab to wearable, real-world applications remains difficult. To overcome issues like low electrical conductivity and limited mechanical robustness, MOFs are often integrated into hybrid composites with conductive nanomaterials. To fully realise the potential of MOF-based wearable sensors, future efforts must improve their stability, lifespan, and biocompatibility while addressing practical concerns such as disposal and performance across diverse environments. Collectively, these advancements position MOFs as versatile platforms for next-generation sensing and drug delivery technologies. Continued research efforts aimed at addressing current challenges are expected to accelerate their clinical translation and widespread adoption. As the field advances, MOF-based wearable systems hold considerable promise for revolutionising disease diagnosis, monitoring and treatment, ultimately contributing to the realisation of personalised health care management.

## 11. Conclusions

Wearable electrochemical glucose sensors based on metal–organic frameworks represent a promising pathway toward non-invasive, continuous glucose monitoring. By leveraging the unique structural and chemical properties of MOFs, researchers have achieved significant advances in sensitivity, selectivity, and device integration. Continued innovation in materials design and system-level engineering, coupled with rigorous clinical validation, will be essential to translate these technologies from laboratory demonstrations to practical healthcare solutions. This review has discussed the fundamental principles of sweat-based glucose monitoring and highlighted the unique advantages of MOFs as advanced functional materials for electrochemical sensing. Their exceptionally high surface area, tunable pore structures, controllable chemical functionality, and abundant active sites, MOFs provide versatile platforms for enhancing analyte adsorption, catalytic activity, enzyme immobilisation, and mass transport. These characteristics have enabled their extensive application in both enzymatic and non-enzymatic glucose sensing systems. The evolution of MOF-based sensors from enzyme-based platforms to nanozyme-based non-enzymatic systems demonstrates the growing ability of MOF materials to address limitations associated with conventional glucose sensing technologies. Enzymatic MOF-based sensors have shown improved enzyme loading protection and catalytic efficiency, while non-enzymatic sensors based on Ni, Co, Cu and multimetallic MOFs exhibited excellent electrocatalytic activity, high sensitivity, low detection limits, and enhanced operational stability. Furthermore, structural engineering strategies such as morphology control, dimensional tuning, defect engineering, and development of bimetallic and trimetallic architectures have significantly improved glucose sensing performance. To overcome the intrinsic conductivity limitations of pristine MOFs, extensive efforts have focused on integrating MOFs with conductive materials, including graphene, carbon nanotubes, metal nanoparticles, conductive polymers and MOF-derived porous carbon. These hybrid systems provide efficient electron transfer pathways while preserving the favourable porosity and catalytic properties of MOFs, resulting in substantial improvements in sensitivity, response time, and long-term stability. Recent advances in flexible electronics have enabled the successful integration of MOF-based sensing materials into wearable platforms. Flexible substrates, textile-based devices, carbon cloth electrodes, and stretchable sensor architectures have demonstrated the feasibility of real-time glucose monitoring under practical conditions. These developments highlight the growing potential of MOF-based wearable electrochemical sensors for personalised healthcare and continuous diabetes management. Collectively, the studies reviewed show that MOFs represent a highly versatile and promising class of materials for wearable electrochemical glucose sensing. Through continuous advances in material design, composite engineering, and wearable device integration, MOF-based sensors are progressively overcoming the limitations of conventional glucose monitoring technologies and paving the way toward next-generation non-invasive biosensing systems.

## Conflicts of interest

There are no conflicts to declare.

## Data Availability

No primary research results, software or code have been included and no new data were generated or analyzed as part of this review.
